# Multiple, Single Trait GWAS and Supervised Machine Learning Reveal the Genetic Architecture of *Fraxinus excelsior* Tolerance to Ash Dieback in Europe

**DOI:** 10.1111/pce.15361

**Published:** 2025-01-17

**Authors:** James M. Doonan, Katharina B. Budde, Chatchai Kosawang, Albin Lobo, Rita Verbylaite, Jaelle C. Brealey, Michael D. Martin, Alfas Pliura, Kristina Thomas, Heino Konrad, Stefan Seegmüller, Mateusz Liziniewicz, Michelle Cleary, Miguel Nemesio‐Gorriz, Barbara Fussi, Thomas Kirisits, M. Thomas P. Gilbert, Myriam Heuertz, Erik Dahl Kjær, Lene Rostgaard Nielsen

**Affiliations:** ^1^ Department of Geosciences and Natural Resource Management University of Copenhagen Frederiksberg Denmark; ^2^ Northwest German Forest Research Institute Hann. Münden Germany; ^3^ Kaunas Forestry and Environmental Engineering University of Applied Sciences Kaunas Lithuania; ^4^ Department of Natural History NTNU University Museum, Norwegian University of Science and Technology (NTNU) Trondheim Norway; ^5^ Lithuanian Research Centre for Agriculture and Forestry Kaunas Lithuania; ^6^ Zentralstelle der Forstverwaltung, Forschungsanstalt für Waldökologie und Forstwirtschaft, Hauptstraße 16 Trippstadt Germany; ^7^ Institute for Forest Biodiversity and Nature Conservation, Federal Research and Training Center for Forests, Natural Hazards and Landscape Vienna Austria; ^8^ Skogforsk, Ekebo 2250 Svalöv Sweden; ^9^ Southern Swedish Forest Research Centre Swedish University of Agricultural Sciences Alnarp Sweden; ^10^ Forest Development Department Teagasc Dublin Ireland; ^11^ Bavarian Office for Forest Genetics (AWG) Teisendorf Germany; ^12^ Institute of Forest Entomology, Forest Pathology and Forest Protection, Department of Ecosystem Management, Climate and Biodiversity BOKU University Vienna Austria; ^13^ Center for Evolutionary Hologenomics, GLOBE Institute, Faculty of Health and Medical Sciences Copenhagen Denmark; ^14^ BIOGECO, INRAE, University of Bordeaux Cestas France

**Keywords:** ash dieback, common ash, disease, GWAS, *Hymenoscyphus fraxineus*, invasive alien pathogen, machine learning, phenology, SNPs, tolerance

## Abstract

Common ash (*Fraxinus excelsior*) is under intensive attack from the invasive alien pathogenic fungus *Hymenoscyphus fraxineus*, causing ash dieback at epidemic levels throughout Europe. Previous studies have found significant genetic variation among genotypes in ash dieback susceptibility and that host phenology, such as autumn yellowing, is correlated with susceptibility of ash trees to *H. fraxineus*; however, the genomic basis of ash dieback tolerance in *F. excelsior* requires further investigation. Here, we integrate quantitative genetics based on multiple replicates and genome‐wide association analyses with machine learning to reveal the genetic architecture of ash dieback tolerance and of phenological traits in *F. excelsior* populations in six European countries (Austria, Denmark, Germany, Ireland, Lithuania, Sweden). Based on phenotypic data of 486 *F. excelsior* replicated genotypes we observed negative genotypic correlations between crown damage caused by ash dieback and intensity of autumn leaf yellowing within multiple sampling sites. Our results suggest that the examined traits are polygenic and using genomic prediction models, with ranked single nucleotide polymorphisms (SNPs) based on GWAS associations as input, a large proportion of the variation was predicted by unlinked SNPs. Based on 100 unlinked SNPs, we can predict 55% of the variation in disease tolerance among genotypes (as phenotyped in genetic trials), increasing to a maximum of 63% when predicted from 9155 SNPs. In autumn leaf yellowing, 52% of variation is predicted by 100 unlinked SNPs, reaching a peak of 72% using 3740 SNPs. Based on feature permutations within genomic prediction models, a total of eight nonsynonymous SNPs linked to ash dieback crown damage and autumn leaf yellowing (three and five SNPs, respectively) were identified, these were located within genes related to plant defence (pattern triggered immunity, pathogen detection) and phenology (regulation of flowering and seed maturation, auxin transport). We did not find an overlap between genes associated with crown damage level and autumn leaf yellowing. Hence, our results shed light on the difference in the genomic basis of ADB tolerance and autumn leaf yellowing despite these two traits being correlated in quantitative genetic analysis. Overall, our methods show the applicability of genomic prediction models when combined with GWAS to reveal the genomic architecture of polygenic disease tolerance enabling the identification of ash dieback tolerant trees for breeding or conservation purposes.

## Summary statement

• *Fraxinus excelsior* or common ash is severely threatened by *Hymenoscyphus fraxineus* the causative agent of ash dieback. Genomic prediction models were combined with GWAS to predict variation in disease tolerance and phenological avoidance.

## 1 Introduction

The threat to indigenous plants from new and emerging forest pests and pathogens has substantially increased in recent decades due to increased international trade and travel. Globalisation has enhanced trade between distant parts of the planet with concomitant exposure of native plants to introduced pathogens, to which they have no evolved resistance or tolerance. This has led to devastating and ongoing pest and pathogen outbreaks such as *Ophiostoma ulmi* and *Ophiostoma novo‐ulmi* causing Dutch elm disease since the start of last century (Brasier 1991), to devastation of ash trees caused by the translocated Emerald Ash Borer (*Agrilus planipennis*) from East Asia to North America possibly from as early as the 1980s (McCullough 2020), to chestnut blight caused by the early 20th century introduction of the fungus *Cryphonectria parasitica* from East Asia to North America, causing the extirpation of the once common American chestnut (*Castanea dentata*) (Anagnostakis 1987). The introduction of pests and pathogens brings novel and strong selection factors to indigenous plants, which in the absence of a shared evolutionary history must solely rely on standing genetic variation to survive and adapt (Budde et al. 2016).

Trees have long generation times (in contrast to associated pests and pathogens), where polygenic mechanisms of disease resistance offer a broad response spectrum to the cornucopia of threats encountered by a tree during its long life (Bruns, Hood, and Antonovics 2015; Yeaman 2022). In forest trees, resistance based on a major gene is therefore likely to be rare (but see the case of western white pine trees against blister rust caused by the introduced fungal pathogen *Cronartium ribicola* in North America (Kinloch et al. 1999)). Polygenic resistance is not only more frequent in nature but offers a more durable form of resistance, which to be defeated requires multiple virulence genes from the pest or pathogen (Bell 1982; Maher 2008; Palloix, Ayme, and Moury 2009).

The development of high‐throughput sequencing technology in combination with improved analytical tools facilitate research on polygenic genetic mechanisms underlying forest tree pest and pathogen resistance. Polygenic traits where multiple loci contribute with small effects to the phenotype, complicate the interpretation of genotype‐phenotype interactions. This is particularly true for Genome Wide Association Studies (GWAS), which have had great success in deciphering the genetic architecture of monogenic traits (Sánchez‐Vallet et al. 2018), but have struggled to unravel the effects of multiple genes in polygenic traits. However, knowledge of the genetic architecture of polygenic disease resistance is crucial to support the stability of future forests in the face of ever growing threats from novel damaging agents (Stocks et al. 2019; Elfstrand et al. 2020).

Ash dieback (ADB), a serious disease of common ash (*Fraxinus excelsior*) in Europe, is caused by the invasive alien ascomycete, *Hymenoscyphus fraxineus* (syn. *H. pseudoalbidus*; basionym *Chalara fraxinea*) (Kowalski 2006; Queloz et al. 2011; Baral, Queloz, and Hosoya 2014). The disease was first observed in north‐eastern Poland in 1992, before appearing in the Baltics in the 1990s and Denmark in 2002, France in 2008, and the UK in 2012 (Marçais et al. 2022). Native to East Asia, *H. fraxineus* is a mild leaf and shoot pathogen of indigenous Asian ash species with insignificant overall impact on its natural hosts (Zhao et al. 2013; Drenkhan et al. 2017). Translocation of *H. fraxineus* to Europe, onto a naïve and compatible host (*F. excelsior*) has initiated the ADB epidemic and resulted in an emerging disease caused by a highly virulent pathogen. Spreading from eastern to western Europe, the range of *H. fraxineus* has no spatial limitations and is only restricted by higher temperatures and low precipitation levels in southern Europe and the occurrence of ash species (Grosdidier et al. 2018; Enderle, Stenlid, and Vasaitis 2019). The disease similarly affects narrow‐leaved ash, *Fraxinus angustifolia*, but crucially *F. angustifolia* has a more southern European distribution where the pathogen is limited by warm‐dry environmental conditions (Dal Maso 2014; Marçais et al. 2022). Disease pressure builds in annual cycles where the wind‐dispersed ascospores of the pathogen spread to new host trees and infect them mainly during the summer months (Marçais et al. 2022). Mycelia of *H. fraxineus* spread from infected leaves into woody parts of the tree causing progressive necrosis and crown dieback, which often lead to mortality of trees of all ages. In late summer and autumn, the infected leaves fall to the ground as leaf litter acting as conduits for sexual recombination, the formation of apothecia and the dispersal of new ascospores, which are completed in the following year.

The impact of ADB on *F. excelsior* is, in addition to tree genotype, dependent on multiple factors including general tree health, age, environmental conditions, infection pressure, presence of other pathogens and endophytes as well as density and spatial heterogeneity of host populations (Grosdidier et al. 2020; Madsen et al. 2021; Marçais et al. 2022). Both tolerance and resistance have been used to describe the variation of damage caused by *H. fraxineus* on *F. excelsior*. In this study the term tolerance of ash to ADB is preferred because the pathogen can fulfil its life cycle on leaves of ash trees with low or high crown damage levels (N.B. for further discussion on tolerance/resistance in relation to ash dieback please see Marçais et al. (2022, 2023)). Previous quantitative genetic studies have revealed a polygenic genetic architecture with moderate to high heritability in ADB tolerance among individual trees. Quantitative genetic studies revealed narrow sense heritability for ADB tolerance of 0.37–0.72 (Pliura et al. 2011; Kjær et al. 2012; Lobo et al. 2014; Muñoz et al. 2016; Seidel et al. 2024) and broad sense heritability of 0.1–0.57 (McKinney et al. 2011; Stener 2013; Enderle et al. 2015; Seidel et al. 2024) among different ash trials in Europe. Furthermore, the level of crown damage due to ADB is genetically weakly correlated to autumn leaf colouring (McKinney et al. 2011; Stener 2013) and significantly correlated to spring bud burst (Stener 2013). Therefore, it has been suggested that the observed genetically determined tolerance may be partly attributed to differences in the timing of phenological stages. Phenological avoidance of severe disease has been identified in other pathosystems such as Dutch elm disease where the susceptibility of elm trees is related to spring phenology (Ghelardini and Santini 2009). However, in previous tests of necrosis formation after direct stem inoculation of *H. fraxineus* onto ash clones and progeny, trees that performed better in the ADB‐affected trials also had reduced necrosis, which indicates defence‐mediated tolerance to ADB; that is, differences in the ability of genotypes to limit the spread of the fungus into and in woody parts (McKinney et al. 2012; Lobo et al. 2015). The relative contribution of defence mediated tolerance and phenological avoidance to overall ash dieback tolerance is unknown.

Recent studies based on GWAS of ash trees covering the UK, Ireland and Germany (Stocks et al. 2019) and Poland (Meger et al. 2024) aimed to identify molecular markers underpinning ADB tolerance. Using binary phenotypes covering the extremes of ash dieback, these studies discovered polygenic tolerance to ADB. The UK study used pooled sequences of 1250 trees and found 61 highly significant SNPs, many of which were within or close to genomic regions known to be related to plant defence. The Polish study used a smaller sample size of 300 trees and found no significant SNPs but revealed genomic loci, which could be used to select individuals for local breeding programs. Here, in contrast to previous GWAS studies, the quantitative nature of ash dieback tolerance was considered, and individual genomic data was used. Individuals were carefully phenotyped on a scale that partly recognises the nature of the tolerance (rather than collapsing phenotypes and genotypes into binary groups, that is, symptomatic vs. nonsymptomatic individuals). This more detailed approach allowed estimation of genetic correlations between phenology traits that are closely related to fitness (frost avoidance) per se, but also have been observed to correlate genetically with ash dieback as discussed above. The present study differs from the Stocks et al. (2019) study by assessing trees aged from 3 to 28 years, rather than young trees (~7 years old) and uses a large sample range that covers a large part of the North, Northeastern, and Central part of the distribution area of *F. excelsior*, sampling across several countries and along ecological gradients. Here, our findings thus rest on a large sample of carefully phenotyped trees in replicated clonal plantings representing a broad genomic background (genotypes were replicated within each site but different sets of genotypes were present at each site). Susceptibility of trees was expressed at different ages and under field conditions based on cooperation between international research organisations in several genetic improvement programs. Given this diversity, the results are expected to be representative of the host‐pathogen interaction in a major part of the natural distribution of *F*. *excelsior*.

Here, we investigated the genetic architecture of *F. excelsior* tolerance to ADB in six countries across Europe, taking advantage of clonal common garden and progeny trials. The aim of the study was to identify associations between the level of crown damage (a continuous trait, which is a proxy for the overall impact of ADB), spring and autumn leaf phenological traits, and molecular markers. Due to the relatively large heritability estimates identified from previous studies and correlations between ADB and phenology, we hypothesised that: (1) genetic variants (SNPs) can predict ash dieback crown damage and phenological variance among individuals; (2) SNPs of importance are located within coding regions and in functional areas related to tree defence and phenology; (3) there are overlaps between SNPs associated with ADB crown damage and SNPs associated with phenology traits. Except for the inclusion of a single progeny trial, ash tree leaves were sampled within clonal trials where multiple ramets of each clone were grown within each site, providing accurate quantitative phenotypes, and therefore providing the best precondition to capture associations between phenotype and genome. To address the above hypotheses, multiple phenotypic traits were recorded on common ash trees on a quantitative scale to reflect the phenotypic spectrum of ADB symptoms on individual trees and of spring and autumn phenology. Furthermore, GWAS were used to identify SNPs associated with phenotypes; subsequently, SNPs with the lowest *P*‐values were used to derive the Random Forests genomic prediction model. Combining these approaches leveraged the power of GWAS to remove low quality SNPs, providing high quality genomic data to the machine learning model.

## 2 Materials and Methods

### 2.1 Field Assessments and Sampling

A unique panel of phenotyped genotypes was constructed by selecting genotypes from a large set of common garden trials (some originally intended for seed collection) with nonoverlapping genotypes located in Austria, Denmark, Germany, Ireland, Lithuania, and Sweden. All trials except one were clonal trials with replicated genotypes, while one of the Danish trials was a progeny trial consisting of 96 half‐sib families (Table S1). The genotypes represented indigenous material from each of the countries at a regional scale (Table S1), which had all been impacted by wind dispersed ascospores of *H. fraxineus* with most trees showing ADB symptoms. The common garden trials had been monitored for ADB symptoms before the present study and significant variation in the intensity of disease symptoms had been observed among genotypes (see references in Table S1). Ash trees with low susceptibility to ADB occur in low frequency across Europe (McKinney et al. 2014), but the applied sampling strategy based on wide sampling and use of prior knowledge from common gardens allowed us to obtain a set of almost 500 genotypes with a gradient from high to low susceptibility to ADB, and also provided a good representation of the *F. excelsior* genetic background in Northern, Northeastern, and Central Europe.

All trees at each trial were assessed for five traits: spring phenology (i.e., spring bud burst), crown damage with typical ADB symptoms, and three scores related to autumn senescence. The traits were scored simultaneously in all sites and descriptive statistics were calculated (Table S1 and Table S2). A total of 4580 trees were assessed across planting sites for the five phenotypic traits, with 841 trees genotyped with microsatellite markers (from the clonal trials to test matching of different ramets of the same genotype) and 486 trees (one per genotype) subjected to whole genome sequencing.

Spring bud burst: The leaf phenological stage of each tree was assessed and scored on a scale from 0 to 7: Score 0: Bud still in winter stage, 1: Bud swollen, black and/or green bud scales enclose the leaves completely, 2: Buds are beginning to burst and leaves only just visible, 3: Leaves visible, very small and have just escaped the bud, 4: Leaves are unfolding, glossy and the shoot starting to stretch, 5: Leaves stretched markedly, still glossy, 6: Leaves full size, still “spring fresh” and not completely hardened, 7: Mature, all leaves are dim and hardened. Please see Figure S1 for illustrations. Each tree was given one score that reflected its average spring phenological stage across the entire crown on the assessment day. Several of the sites were scored twice (Table S1) to assure capturing a time point with maximal phenotypic variation among genotypes.

ADB crown damage: Trials with young trees were scored on a scale with five categories: 0: No visible symptoms, 1: < 10% of the crown with ADB symptoms, marginal damage on stem and crown, 2: 10%–50% of the crown with ADB symptoms, presence of dead parts in the crown and discoloured necrotic parts on stem, 3: > 50% of the crown with ADB symptoms, prominent to highly dominant presence of damage on shoots, branches and stem, including larger necrotic areas on stem and branches, 4: tree dead because of severe infection. For older trees three additional categories were included (see Table S3). To rank genotypes across the sites, crown damage scales were transformed into percentage using the following five class means (0%, 5%, 30%, 75%, 100%).

Autumn leaf yellowing: This trait was scored on a five‐step scale reflecting the overall impression of the autumn colour of each tree using the following scale: 0: leaves still dark green, 1: leaves slightly lighter, or dark green but with yellowing leaf nerves, 2: leaves green but with yellow spots on leaflets, 3: yellowing leaflets, 4: completely yellow leaves. See Table S3 and Figure S2 for detailed descriptions and illustrations.

Autumn leaf loss: This trait was scored as percentage of leaves lost in the crown apparently due to autumn senescence and scored in 10% classes (Table S3 and Figure S2).

Autumn status: This trait reflects the overall progress towards dormancy status of a tree; the remaining leaves showing senescence symptoms combined with the progression of senescence symptoms. Symptoms of senescence of foliage: summer green colour becomes lighter, yellowing, brownish, withering (typically from the rim towards the middle) and crusted, dried out or hanging leaves. Thus, the score is gradual and shows average tree senescence level (full dormancy = 100%) (Table S3 and Figure S1).

A subset of *F. excelsior* individuals which had been phenotypically assessed, were sampled from each site for DNA extraction. Based on data of ADB assessments from previous years we aimed to span the entire ADB crown damage range (healthy, intermediate and unhealthy) from each trial when selecting the trees. Information regarding phenotypic assessment of trees at each site and those selected for DNA sequencing is presented in Table S2. From each sampled tree, 2–4 fresh leaflets were collected and placed in ziplock‐bags with silica gel. The samples were stored at 4°C until further processing.

### 2.2 Statistical Analyses

Phenotypic data for the GWAS was derived from the ADB crown damage and phenology scores described above. Least square mean values (LSMeans) were estimated for each genotype (for genotypes from clonal trials as clonal LSMeans, for genotypes from progeny trial as family LSMeans) per trial using the model given below using PROC GLM in SAS 9.4:

$$Y_{ijk}=µ+B_{i}+C_{j}+\varepsilon_{ijk},$$

where *Y_ijk_* is the phenotypic score measured for trees, *µ* is the overall mean of the trial score, *B*_*i*_ is the fixed effect of block within a trial, *C*_*j*_ is the random effect of clone/family, and *ε*_*ijk*_ is the residual.

Since the genotypes were phenotyped at different sites and ages, the LSMeans had to be converted to relative values before they could be used in the GWAS. We therefore subtracted the mean value per trial as follows:

$${\text{rPhenotype}}_{j(z)}={\text{LSMean}}_{jz}–{\text{LSMean}}_{z},\text{for}\;\text{genotype}\;j,\;\text{tested}\;\text{in}\;\text{trials}\;z.$$

Thus rPhenotype_*j(z)*_ quantifies how much a given phenotype deviates (positively or negatively) from the average tested genotype at its site. We used this value as a quantitative measure in the GWAS to identify SNPs that contributed to a better or poorer performance of the single genotype in the given trait.

Genetic correlations (*r*_*G*_) among traits were calculated using bivariate analysis in ASReml v4.2 using the following equation:

$$r_{G}=\frac{\sigma \mathrm{ij}}{\sigma i\sigma j},$$

where *σij* is the genotype covariance component between traits *i* and *j*, *σi* and *σj* are the standard deviations for genotype variance components for traits *i* and *j*, respectively. Again, this was done per trial since the genotypes were nonoverlapping between sites.

### 2.3 DNA Extraction

DNA was extracted from 20 mg of dried leaf tissue using the NucleoSpin Plant II kit (Macherey‐Nagel, Denmark). DNA was extracted according to the manufacturer's protocol with the following modifications: 100 μL of lysis buffer PL1 was added to each sample, an additional initial physical disruption step using a Qiagen Tissue Lyser II (Qiagen, Germany) at 25 rpm/s for 1 min with two 3 mm steel beads for each sample was made, then 500 μL additional lysis buffer PL1 was added to each sample, before two additional physical disruption steps at 25 rpm/s for 1 min. Subsequently, 10 μL RNase A was added to each tube, the samples were vortexed briefly and incubated at 65°C for 15 min and inverted two–three times during the incubation. Samples were placed in a centrifuge at 8000 rpm for 5 min to remove crude lysate. Supernatant was transferred to a violet ringed NucleoSpin ii tube, and from this point extracted according to the manufacturer's instructions. Extracted DNA was eluted in 50 μL volumes and concentration was measured using a Qubit 3.0 fluorometer (Invitrogen, USA) and Qubit dsDNA BR kit (Invitrogen, USA). In total, 486 trees were selected for whole genome sequencing.

### 2.4 Microsatellite Analysis

Before library preparation at least two ramets per genotype within each clonal trial were genotyped using microsatellite markers to omit potential grafting or sampling errors in the data. In cases of mismatch between genotyped ramets, the genotype was omitted from the study. Microsatellite analyses were performed with three selected primer pairs multiplexed in a mix: FEMSATL11 and FEMSATL19, as well as FEMSATL12 (Lefort and Douglas 1999; Gerard, Fernandez‐Manjarres, and Frascaria‐Lacoste 2006). PCR amplifications were carried out using the Qiagen Multiplex PCR Kit (Qiagen, Germany) according to the manufacturer's instructions but scaled down to 10 μL reaction volume. PCR amplifications were performed under the following conditions: initial denaturation at 95°C for 15 min, 30 cycles of denaturation at 94°C for 30 s, annealing at 57°C, extension at 72°C for 60 s, and final extension at 60°C for 30 min. PCR products were analysed on the ABI 3130xl Genetic Analyser (Applied Biosystems, Foster City, CA, USA).

### 2.5 Library Preparation

DNA was sheared using a LE220‐plus Focused‐ultrasonicator (Covaris, USA), with a target insert length of 500 bp. Sheared DNA fragment size and concentration was assessed using a Fragment Analyzer 5300 with 48 capillaries. The Fragment Analyzer was operated by the National High‐Throughput DNA Sequencing Centre (University of Copenhagen, Denmark). DNA libraries were built using the BEST protocol (Carøe et al. 2018) with an input DNA quantity of 100 ng. Prepared libraries were PCR‐indexed (Table S4) using AmpliTaq Gold polymerase with the following conditions: 95°C for 10 min, followed by 12–16 cycles at 95°C for 20 s, 60°C for 30 s, 72°C for 40 s, and a final elongation step at 72°C for 7 min. DNA libraries were dual indexed and subsequently pooled into equimolar concentrations with a maximum of 96 samples in each pool.

Pooled DNA was sequenced by Macrogen Europe (Amsterdam, The Netherlands) on the NovaSeq. 6000 platform with 150 bp PE reads, using the S4 flow cell workflow. A further 10 samples were sequenced on the Illumina HiSeq. 4000 platform at the National High‐Throughput DNA Sequencing Centre (University of Copenhagen, Denmark). All sequences were demultiplexed using bcl2fastq v2.20.0.422. Demultiplexing was performed by Macrogen Europe and the National High‐Throughput DNA Sequencing Centre.

### 2.6 Adaptor Trimming, Quality Control and Mapping

Quality control and adaptor removal of demultiplexed DNA was performed using BBMap v38.22 (Bushnell 2014). Adaptors were trimmed with BBduk and sequences were filtered for low quality reads, removing those with average quality less than 20, and further filtered using the following options mink = 11, qtrim = rl, minlen = 50, tbo = T, ktrim = r, and *k* = 23. Remaining sequences were aligned to the *Fraxinus excelsior* reference genome (BATG0.5) (Sollars et al. 2017), and converted to SAM format using BWA v0.7.17‐r1188 (Li and Durbin 2009), and to BAM format using Samtools v1.10 (Li et al. 2009). Sequenced reads, which did not align to the *F. excelsior* reference genome and read pairs which mapped to mitochondrial and chloroplast regions were removed from the analysis. Remaining sequences that mapped to the nuclear genome gave a mean sequencing depth of 9x with a standard deviation of 8.4 (Table S5).

### 2.7 Genotype Likelihoods, Imputation, and Genotype Probabilities

**Figure 1:**
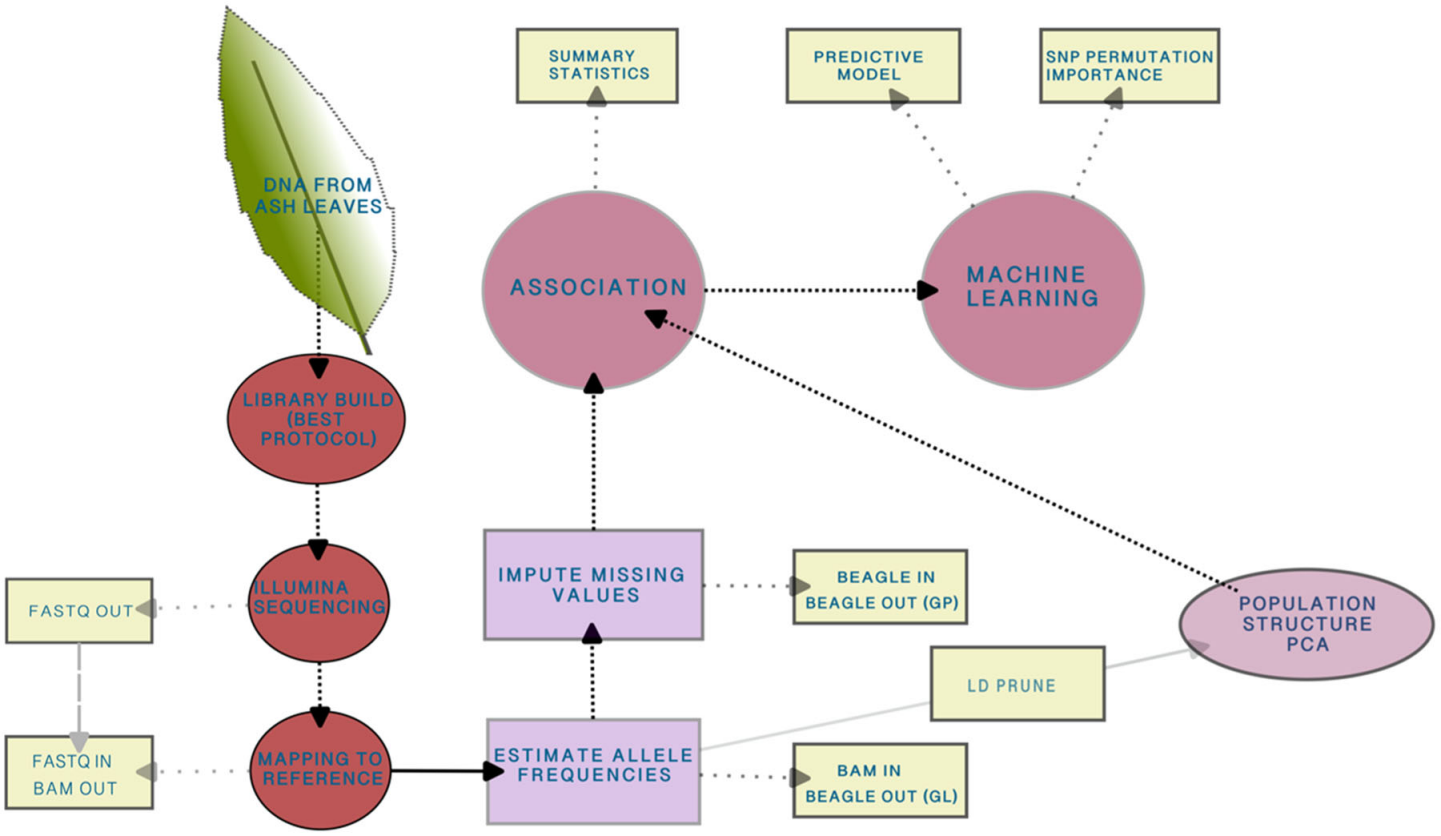
Flow chart of laboratory work and computational pipeline. After collecting *Fraxinus excelsior* leaves, DNA was extracted from a leaflet, sequenced, and analysed using GWAS and machine learning. GL = genotype likelihoods, GP = genotype probabilities, LD = linkage disequilibrium, PCA = principal component analysis.

Genotype likelihoods were calculated for polymorphic positions that passed quality control checks in the *F. excelsior* reference genome (SNP sites) within ANGSD v0.935 (Korneliussen, Albrechtsen, and Nielsen 2014). The likelihoods were calculated using the SAMtools model (gl 1) and a minor allele frequency threshold (MAF) of 0.05; sites with data for fewer than 30 individuals were removed (minInd); a minimum base quality score of 20 (minQ) was used; excessive mismatches were removed (−C 50); reads with a flag above 255 (remove_bads 1) and read pairs, which did not map correctly were removed (only_proper_pairs 1); reads that had multiple best hits were removed (uniqueOnly 1); finally a *p* value threshold of 2 × 10^−^^6^ was used to call SNPs. Furthermore, the BAQ algorithm was included in the analysis to remove false SNP calls due to misalignment of indels (Li 2011). To reduce computational time ANGSD was launched in parallel, using GNU parallel v20220422 (Tange 2022). Due to the varying level of coverage between samples of aligned whole genome sequences, missing values were imputed, and subsequently genotype probabilities were calculated in Beagle v3.3.2 (Browning and Browning 2007). An overview of the computational pipeline is shown in Figure 1.

### 2.8 Population Structure

Population structure was inferred using SNP genotype likelihoods from ANGSD and assessed using PCAngsd v0.97 (Meisner and Albrechtsen 2018). Before calculation of principal components, linked SNPs were pruned using Plink2 v1.90beta6.24 (Purcell et al. 2007), and the indep‐pairwise setting was applied, with a window size of 50, a variant count of 0.5 and a pairwise *R* ^2^ value of 0.5. To prevent confounding factors arising during subsequent association tests, a covariance matrix was estimated and included in subsequent association tests. Eigenvectors were calculated in R from the covariance matrix using the R base function ‘eigen’. Significance values of eigenvectors were calculated using the Tracy‐Widom test within the R package AssocTests (Wang, Zhang, and Li 2020). There were three significant principal components (eigenvectors) which were included as covariates in association tests.

### 2.9 Association Testing

We performed a genome wide association analysis for all quantitative traits using a generalised linear framework implemented in ANGSD v0.935 (Skotte, Korneliussen, and Albrechtsen 2012). The quantitative phenotype used in the association was the deviation of the genotype from the average performance at the site it was tested (rPhenotype_*j(z)*_, as described above). The following options were selected for the association test, ‘doAsso 6’ as the dosage model was used, ‘yQuant’ as phenotypes were quantitative, ‘Pvalue 1’ to print *p* values, ‘doMaf 4’ to estimate major/minor allele frequency from genotype probabilities, ‘minHigh 30’ to require a minimum of 30 credible genotypes, ‘minCount 30’ to require a minimum of 30 credible minor alleles and the ‘cov’ option which contained the first three covariates from the PCAngsd output. Resultant *p* values were ranked (the lowest *P*‐value SNP was ranked as number 1) and used as input to a machine learning model. SNPs were not corrected for multiple tests, as after ranking based on *p* value they were used as input for genomic prediction with Random Forests. An association for each genotype and phenological trait was created independently.

### 2.10 Genomic Prediction Using Random Forests

The SNPs with the lowest *p* value from each trait in ANGSD association testing were exported to an R implementation of Random Forests (v4.6‐14) for a genotype‐phenotype linear regression analysis. Before running the model, SNPs were called and converted to HapMap format. That is, top ranked SNPs based on *p* value were converted from Beagle format containing genotype probabilities to VCF using FCgene v1.0.7 (Roshyara and Scholz 2014), then SNPs were called and converted to HapMap format using a custom script available here: https://github.com/clydeandforth/gwas_ash_adapt. SNPs were used as dependent variables, representing the genotype, and the phenotypic traits were independent variables represented by a continuous scale. The random forest ensemble model was conducted for each phenotypic trait independently. Within each model, hyperparameter settings such as *mtry* and *ntree* were tested and settings, which provided the most stable model, were used.

Genetically distinct genotypes were randomly split 50%:50% into training (*n* = 243) and test (*n* = 243) datasets. Both the training and test datasets contained the genotypic and phenotypic data for 50% of the samples. The model was trained on the training data set and then used to predict the phenotypic score of the test data set. The linear relationship between the predicted score and the actual phenotypic score was predicted using an adjusted *R* ^2^ value. To understand the ability of varying quantities of SNPs to predict the phenotype (adjusted *R* ^2^), SNPs were divided into groups (cohorts) by lowest *p* value, that is, the lowest *p* value was SNP rank 1 and the 10th lowest was SNP rank 10. Based on these rankings, the SNPs were allocated into cohorts of the lowest 10, 50, 100, 500, 1000, 10 000, 25 000, 50 000, 100 000 and 1 000 000. For each cohort a train/test‐run was conducted 100 times (using a different seed number for each run, thereby giving a unique split of the data in each run). Conducting the train/test‐run 1000 times did not change the results. The mean *R* ^2^ of each permutation set for each cohort was taken as the final adjusted *R* ^2^ value, and the highest adjusted *R* ^2^ value indicated the cohort of SNPs that best predicted the phenotypes (best cohort).

### 2.11 Functional Enrichment Analysis

The best performing cohort of SNPs from the machine learning models were aligned to the *F*. *excelsior* reference genome, and structural annotations of genic regions from the alignment were extracted. Structural annotations containing SNPs in FASTA amino acid format were used as input to the KOBAS‐i online webserver (Bu et al. 2021). The organism most closely related to *F*. *excelsior* in the KEGG pathway database was *Olea europaea* var. *sylvestris* which was used as a reference for pathway overrepresentation analysis. Significantly overrepresented functional groups were calculated using Fisher's exact test.

### 2.12 Permutation Importance and Feature Selection

The predictive ability of each SNP within the maximal cohort for each phenotypic trait (i.e., the number of SNPs which gave the highest *R* ^2^ value) was evaluated using the importance function in Random Forest, by selecting type = 1 and scale = FALSE. The value assigned to each SNP as a feature is the permutation importance (PI) (Debeer and Strobl 2020). The PI is a numeric value which can be positive or negative; a higher PI value indicates higher predictive power. To account for linkage disequilibrium (LD), SNPs were pruned as described above using Plink v1.90beta6.24 (Purcell et al. 2007), as LD can considerably affect importance measure calculations (Meng et al. 2009). The final SNP sets for each trait used in permutation importance calculations are presented in Supporting Information Data S1–S5.

## 3 Results

### 3.1 Genetic Correlation Between Ash Dieback Crown Damage and Phenological Traits

Our data shows that estimates of genetic correlation between ADB crown damage and phenology vary substantially among clonal trial populations, but autumn leaf yellowing was consistently and significantly negatively correlated with crown damage in all trials, except Ireland which showed the same trend but lacked significance (Table 1). This negative correlation means that the more a tree shows leaf yellowing at a given timepoint, the less it is affected by ash dieback. Genetic correlations for the three remaining phenotypic traits were variable, as autumn status showed a significant negative correlation to ADB crown damage at three of the sites, autumn leaf loss showed a significant positive correlation to ADB crown damage in three of the trials assessed and spring budburst had both positive and negative correlations to ADB crown damage. Due to the consistent significant negative correlation between ADB crown damage and autumn leaf yellowing, genomic associations for these traits are presented below. Data for the remaining three traits are mainly presented in supplementary information.

**Table 1:** Genetic correlations between ADB crown damage and phenological traits in *Fraxinus excelsior*. Correlations in bold are significant.

Sampling site	Autumn leaf yellowing	*±SE*	Autumn leaf loss	*±SE*	Autumn status	*±SE*	Spring bud burst	*±SE*
**Kusel, Germany**	**−0.17**	***0.15***	−0.13	*0.15*	−0.002	*0.21*	−0.01	*0.14*
**Chiemsee, Germany**	**−0.27**	***0.22***	**0.33**	***0.20***	0.19	*0.22*	0.18	*0.24*
**Landstuhl, Germany**	**−0.24**	***0.21***	−0.02	*0.21*	0.16	*0.19*	**0.22**	***0.21***
**Tjærby, Denmark**	**−0.69**	***0.36***	−0.04	*0.13*	**−0.22**	***0.16***	**−0.54**	***0.13***
**Tuse Næs, Denmark**	**−0.56**	***0.17***	0.18	*0.22*	**−0.40**	***0.18***	0.06	*0.26*
**Silkeborg, Denmark**	**−0.69**	***0.68***	−0.10	*0.60*	**−**0.47	*0.56*	0.49	*0.50*
**Snogeholm L, Sweden**	**−0.71**	***0.14***	−0.02	*0.18*	**−0.99**	***0.14***	−0.09	*0.21*
**Snogeholm S, Sweden**	**−0.54**	***0.18***	**0.61**	***0.13***	−0.11	*0.27*	**−0.27**	***0.18***
**Kilmacurragh, Ireland**	−0.09	*0.12*	**0.35**	***0.12***	0.02	*0.14*	**−0.37**	***0.11***
**Šakiai, Lithuania**	NE	*NE*	0.21	*0.23*	0.26	*0.26*	−0.32	*0.29*
**Feldkirchen, Austria**	**−0.41**	***0.25***	0.07	*0.40*	−0.06	*—*	**−0.44**	***0.18***

*Note:* Significance threshold: *p* ≤ 0.05.

Abbreviations: NE, nonestimable due to lack of genetic variation among clones; SE, standard error.

### 3.2 Genotype‐Phenotype Associations Reveal Signatures of Ash Dieback and Autumn Leaf Yellowing Within Genomic Linkage Blocks

**Figure 2:**
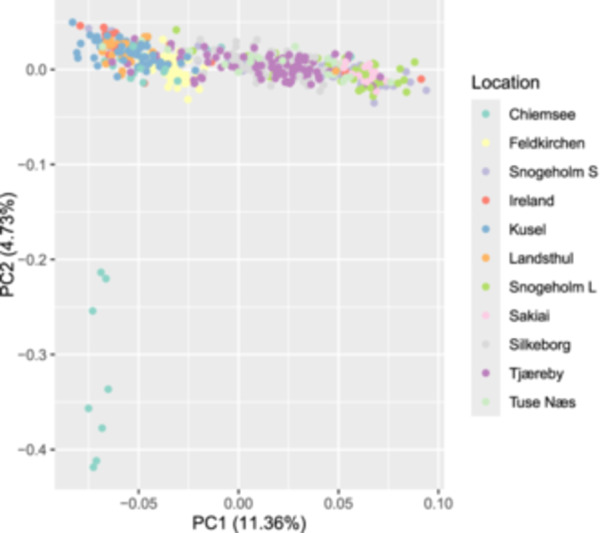
Principal component analysis showing clusters of sequenced *Fraxinus excelsior* individuals. Sequenced individuals are grouped according to their sampling site. [Color figure can be viewed at wileyonlinelibrary.com]

**Figure 3:**
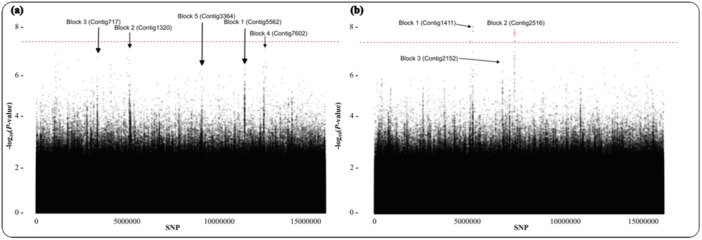
Manhattan plots of ash dieback crown damage (a) and autumn leaf yellowing (b). The plots reveal a polygenic genetic architecture and shown in the plots are several linkage blocks and their contig number in the *Fraxinus excelsior* BATG‐0.5 draft genome. SNPs marked in red are below the standard GWAS threshold of *p* ≤ 5 × 10^−^^8^ which is also marked with a broken red horizontal line. [Color figure can be viewed at wileyonlinelibrary.com]

Associations between genomic variants (SNPs) and phenology traits were tested for significance. Within the GWAS workflow the population structure was accounted for by including principal components as covariates. The population structure of all sequenced samples shows that they largely cluster geographically by their sampling site (Figure 2). Across phenological traits SNPs followed a polygenic genetic architecture (Figure 3). This is consistent with previous plant GWAS (Demirjian et al. 2023). Linkage blocks displayed using Manhattan plots revealed associated SNPs (Figure 3). There were no SNPs below the typical GWAS significance threshold of *p* ≤ 5 × 10^−^^8^ for the ash dieback crown damage association (Figure 3a) and eight SNPs below the threshold for the autumn leaf yellowing association (Figure 3b). None of the eight SNPs significantly associated with autumn leaf yellowing were located within or near to coding regions. The top ranked SNPs, that is, those with the lowest p values, were used as input data for Random Forest linear regression model.

### 3.3 High Phenotypic Variance is Predicted by a Relatively Low Number of SNPs

**Figure 4:**
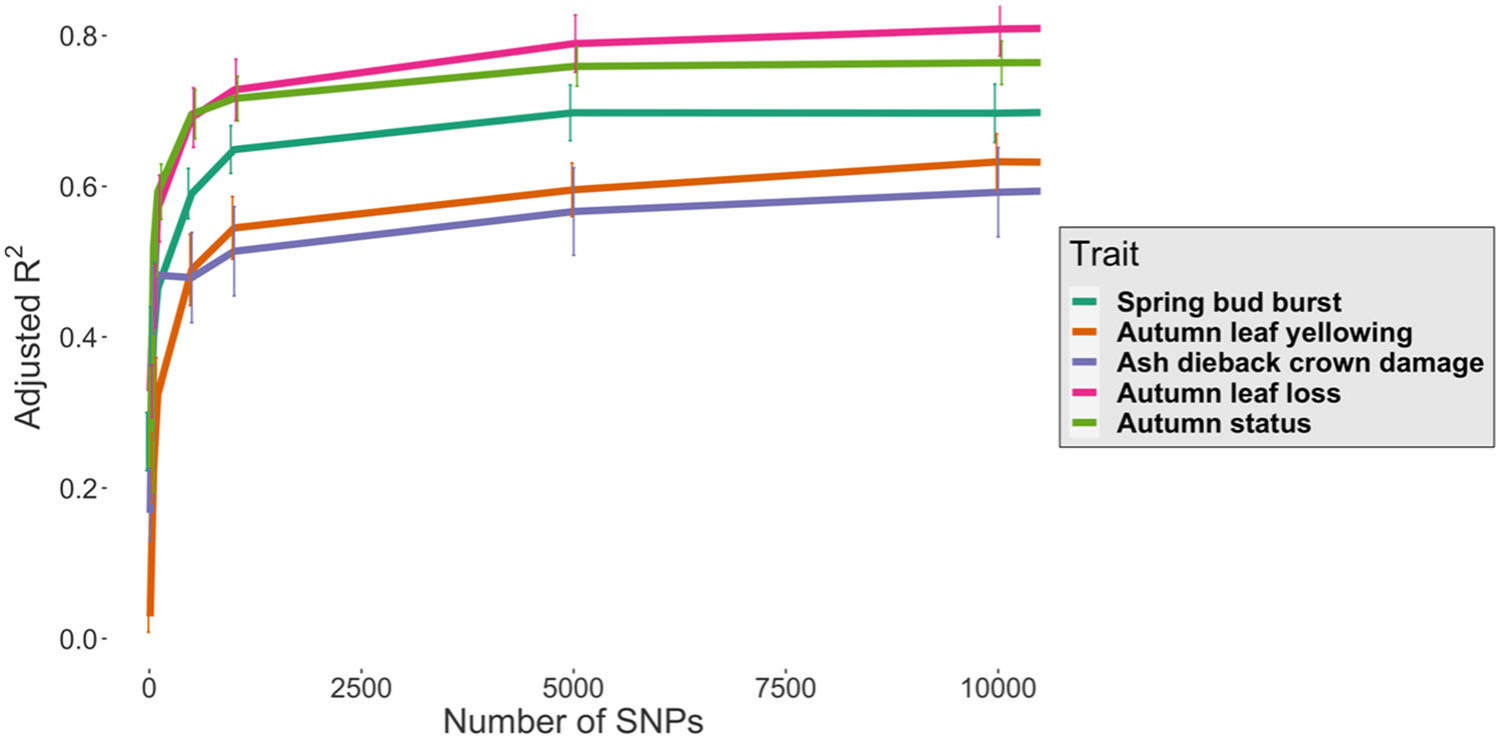
Average adjusted *R* ^2^ for five phenology and ash dieback crown damage traits using the top 10–10 000 ranked SNPs from Random Forests machine learning models. The observed phenotype for five traits was predicted using genotypes (SNPs) with an adjusted *R* ^2^ value between 0 and 1 (displayed in the *y*‐axis). [Color figure can be viewed at wileyonlinelibrary.com]

SNPs were fitted into a Random Forest linear regression model where the maximum adjusted *R* ^2^ value for each trait was calculated by placing SNPs into cohorts between 10 and 1 million based on their GWAS *p* value, with for example cohort 10 being the SNPs with the 10 lowest *p* values for that trait association. This approach revealed that the maximal number of SNPs with high predictive ability for observed genotypes (adjusted *R* ^2^) was 25k (adjusted *R* ^2^ = 64%) for ADB crown damage, 100k (adjusted *R* ^2^ = 75%) for spring bud burst, 10k (adjusted *R* ^2^ = 63%) for autumn leaf yellowing, 100k (adjusted *R* ^2^ = 84%) for autumn leaf loss and 50k (adjusted *R* ^2^ = 78%) for autumn status (Figure 4). High predictive power as indicated by adjusted R^2^ values for each trait were reached using far lower numbers of SNPs. For example, 50 SNPs predict 45% of the genotypic variance for ADB crown damage, increasing to 48% for 100 SNPs to 57% for 5k SNPs before the maximal *R* ^2^ of 64% is reached with 25k SNPs. Similarly, the pattern of genomic prediction in Figure 4 for the autumn leaf yellowing trait shows a more gradual rise to reach the plateau, as the first 10 SNPs have an adjusted *R* ^2^ value of 3% which increases to 32% with 100 SNPs and 54% with 1k SNPs before reaching a peak of 63% at 10k SNPs. The remaining three traits (spring bud burst, autumn leaf loss and autumn status) required more SNPs to reach their maximal adjusted *R* ^2^ value at 50k (autumn status) and 100k (spring bud burst and autumn leaf loss) SNPs, and as with the ADB crown damage trait have a rapid increase in proportion of variance explained from relatively few SNPs (Figure 4). For all traits, increasing the number of SNPs using those selected by GWAS *p* value enables greater prediction of phenotypic variance (Figure 4).

The cohort of SNPs resulting in the highest adjusted *R* ^2^ value was used for functional enrichment and permutation importance (PI) analysis. Permutation importance is a measure of model performance where each feature (here SNPs) is randomly shuffled, and the error rate of the model calculated (Nicholls et al. 2020). For example, as 25k SNPs produced the maximal adjusted *R* ^2^ value for ADB crown damage, SNPs from this cohort were first assessed for enrichment of the gene function based on their loci. Secondly, the SNP set was pruned for linkage disequilibrium and the reduced number of SNPs were thereafter ranked according to their permutation importance. Selected SNPs were those that were top ranked from a machine learning model of PI (i.e., having the greatest effect on phenotype prediction). SNPs which remained after pruning were the final list of SNPs for each trait and those which were used for further analysis (Supporting Information Data S1–S5).

### 3.4 SNPs Which Predict ADB Crown Damage Are Located Within Coding Regions of Genes Related to Stress Tolerance and Phenology

Ash dieback crown damage is an overall measure of the damage inflicted on woody parts of individual trees by *H*. *fraxineus* over several years. Multiple ramets of each genotype were assessed to obtain an average figure. Genomic predictions of ADB crown damage using SNP variants gave the highest predictive effect using 25k SNPs (adjusted *R* ^2^ = 63%). After pruning for linkage disequilibrium, a final list of 9155 SNPs remained, and the top 100 ranked unlinked SNPs could predict 55% (adjusted *R* ^2^ = 55%) of the variation. These SNPs were ranked based on their PI score. The loss of predictive ability after removing linked SNPs was negligible as the unlinked 9155 SNPs still had the same value (when digits were rounded), with an adjusted *R* ^2^ of 63%. The linked SNPs which were pruned from the data set had similar predictive ability with a marginally reduced *R* ^2^ of 62%, revealing redundancy among the 25k SNPs which is likely due to linkage disequilibrium.

**Figure 5:**
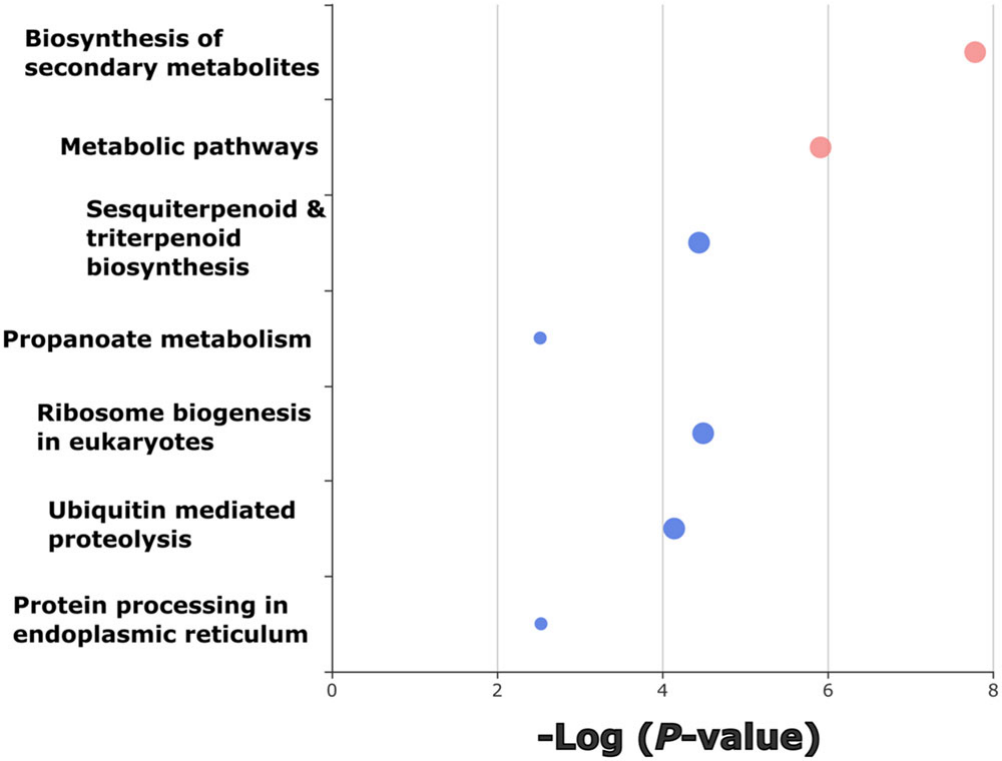
Enrichment analysis of genomic regions comprising the 25k single nucleotide polymorphisms (SNPs) with the highest predictive ability within a machine learning model for ash dieback crown damage. Colours represent related functional groups (in this figure only pink/red circles represent related functional groups), as pathways were calculated using a network algorithm (Rosvall and Bergstrom 2008). Note that blue circles indicate unrelated functional groups. Bubble size represents the magnitude of enrichment for each functional group. Displayed bubbles have a *p* ≤ 0.05. [Color figure can be viewed at wileyonlinelibrary.com]

Alignment of the 25k SNPs to gene loci on the ash reference genome and a subsequent clustering analysis, provided a profile of enriched functional groups with high predictive power of ADB crown damage (Figure 5). Among the enriched functional homologues were those groups encoding biosynthesis of secondary metabolites and sesquiterpenoid and triterpenoid biosynthesis. These enriched defence‐related functional groups indicate coding variation in tolerance and phenology genes, which may reflect the observed phenotypic variation in tolerance. For example, the biosynthesis of secondary metabolites group includes phytoalexins and phenolics, known antimicrobial, communication and stress‐response compounds (Bhattacharya, Sood, and Citovsky 2010; Ahuja, Kissen, and Bones 2012). This indicates a variable defence response among *F. excelsior* individuals to *H. fraxineus*.

**Figure 6:**
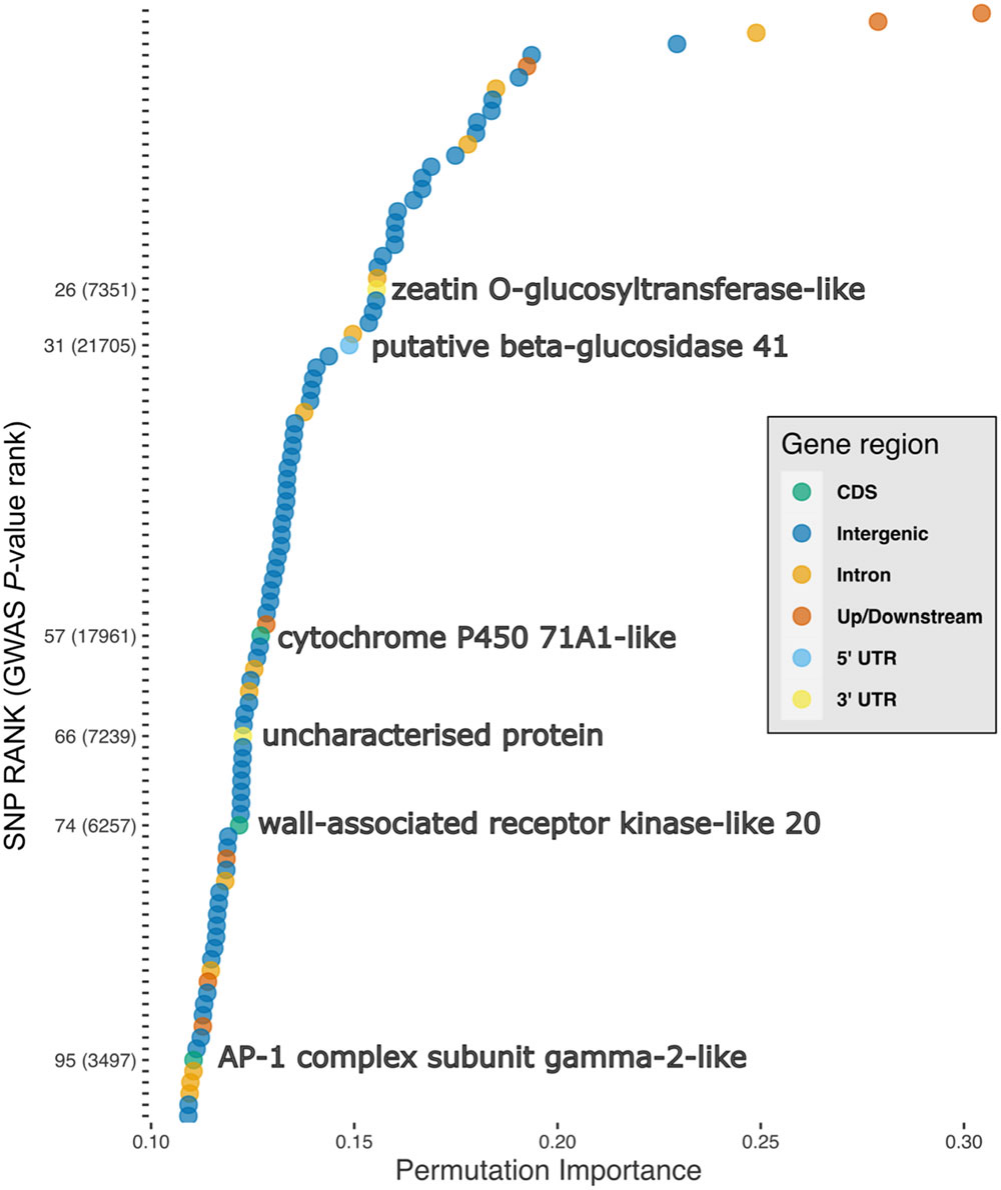
Top 100 SNPs ranked by permutation importance linked to ash dieback crown damage in *Fraxinus excelsior* individuals. SNPs were ranked from a panel of 9155 SNPs, ranked according to their permutation importance. SNPs are marked according to their position within a genic region: SNPs within 500 bp of genic regions are marked ‘Up/Downstream’; SNPs resulting in a coding domain substitution (CDS) are shown in green and the value given in the *y* axis is their PI rank with GWAS rank in parentheses. SNPs which cause missense mutations have functionally annotated coding domains. Permutation importance score is shown in the *x* axis. [Color figure can be viewed at wileyonlinelibrary.com]

The PI score and genomic location (i.e., within a coding domain, intragenic region, up or downstream of a gene, intergenic region or untranslated region) of the top 100 ranked SNPs is shown in Figure 6. SNPs within the top 100 for PI score which were located on or near to genes were functionally annotated using the NCBI and KEGG databases. Among the top 100 SNPs, three were on coding domains; all three SNPs cause non‐synonymous, missense mutations which change the translated amino acid (Table 2). In addition three SNPs were located in untranslated regions (SNP 2, telomerase reverse transcriptase; SNP 6, presenilin At1g08700; SNP 56 disease resistance RPM1‐like) and although these are not translated into proteins they have important effects on transcriptional regulation, such as mRNA stability and translation which have demonstrable effects in human disease (Hindorff et al. 2009; Steri et al. 2018; Conteduca et al. 2021). Of the remaining top 100 SNPs, seven were located up or downstream of a gene, 13 were within intragenic regions (introns), and the remaining 74 were within intergenic regions.

**Table 2:** List of nonsynonymous SNPs with high permutation importance, located within functionally important genes.

SNP	Base change (major‐ minor allele)	Amino acid change	SNP Effect	MAF	Gene ortholog	Trait
Contig6850_25947	A‐C	Leucine ‐ Phenylalanine (L‐F)	A ‐ linked to tolerance	0.34	cytochrome P450 71A1‐like	ADB‐CD
Contig2195_24284	G‐T	Arginine ‐ Leucine (R‐L)	T ‐ linked to tolerance	0.16	wall‐associated receptor kinase‐like 20	ADB‐CD
Contig216_50416	A‐G	Valine ‐ Isoleucine (V‐I)	A ‐ linked to tolerance	0.34	AP‐1 complex subunit gamma‐2‐like	ADB‐CD
Contig3363_3634	T‐A	Asparagine ‐ Tyrosine (N‐Y)	T ‐ linked to early senescence	0.35	putative pectinesterase/pectinesterase inhibitor 45	ALY
Contig42_161402	T‐C	Glutamine ‐ Arginine (Q‐R)	T ‐ linked to early senescence	0.1	CBS domain‐containing CBSX1, chloroplastic‐like	ALY
Contig3270_1675	G‐C	Leucine ‐ Valine (L‐V)	G ‐ linked to late senescence	0.25	l‐type lectin‐domain containing receptor kinase IV.2‐like	ALY
Contig5588_26554	C‐A	Histidine (H)‐ Asparagine (N)	C ‐ linked to late senescence	0.23	CTL‐like protein DDB_G0274487	ALY
Contig4620_41903	T‐G	Isoleucine ‐ Arginine (I‐R)	T ‐ linked to early senescence	0.55	MDIS1‐interacting receptor like kinase 2‐like	ALY

Abbreviations: ADB‐CD, ash dieback crown damage; ALY, autumn leaf yellowing.

The highest ranked SNP within a coding domain was the 57th ranked SNP overall by PI and by GWAS ranking had the 17961st lowest *p* value (Figure 6). This SNP was located within a gene region homologous to a cytochrome P450‐71A1‐like protein which is involved in avocado fruit ripening (Bozak et al. 1990), but in which a mutation on a gene homologue in barley suppresses the development of necrotrophic fungal pathogens through reinitiating programmed cell death, a plant defence mechanism that reduces access to nutrient rich plant tissues and which can be hijacked by pathogen elicitors (Ameen et al. 2021). The second of the three SNPs is located within a region encoding a wall‐associated receptor kinase‐like 20 protein; homologues of this gene are part of the pattern triggered immunity (PTI) system, a conserved plant defence system which recognises conserved components of plant pathogens and triggers an immune response (Jones and Dangl 2006). The third and final coding domain SNP was located on an AP‐1 complex subunit gamma‐2‐like gene which in *Arabidopsis* is critical for the development of male and female gametophytes (Zhou et al. 2022).

### 3.5 Autumn Leaf Yellowing ‐ Pathogen Triggered Immunity and Phenological Regulation Are Linked to Trait Variation

Autumn leaf yellowing is a quantitative measure of loss of green pigment in leaves. Colour change in *F. excelsior* is usually green to yellow, but leaves may stay green before falling in Central Europe (Roloff and Pietzarka 1997). Genotypic prediction of autumn yellowing based on the top 10k ranked associated SNPs could predict 63% of this trait. After LD pruning there were 3740 SNPs remaining. This increased the *R* ^2^ to 72% suggesting that some pruned SNPs did not add to the predictive ability but instead decreased the accuracy of the model and did not contribute to an accurate genotype prediction. However, the removed linked SNPs (*n* = 6260) gave a *R* ^2^ of 58% suggesting that many SNPs were linked and similar to ADB crown damage reveals redundancy in the larger (10k SNP) data set.

**Figure 7:**
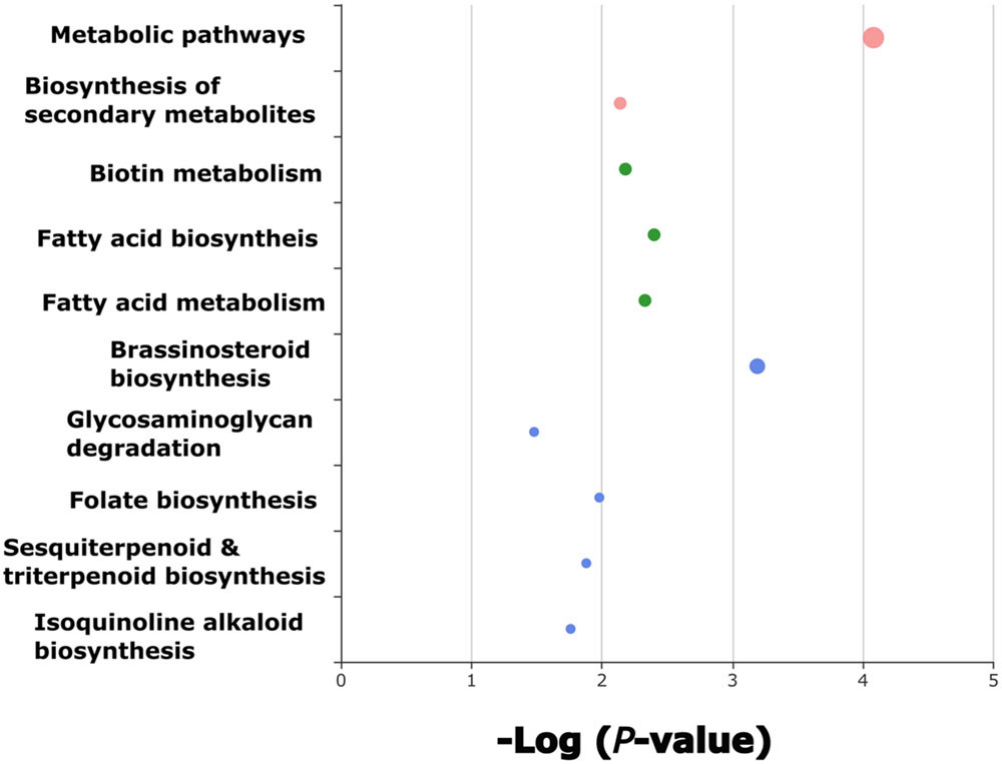
Enrichment analysis of genomic regions comprising the 10k single nucleotide polymorphisms (SNPs) with highest predictive ability within the machine learning model for autumn leaf yellowing in *Fraxinus excelsior* individuals. Colours represent related functional groups, as pathways were calculated using a network algorithm (Rosvall and Bergstrom 2008). Note that blue circles indicate unrelated functional groups. Bubble size represents the magnitude of enrichment for each functional group. Displayed bubbles have a *p* ≤ 0.05. [Color figure can be viewed at wileyonlinelibrary.com]

Enrichment analysis based on the 10k SNP data set reveals functional groups homologous to plant defences such as brassinosteroid biosynthesis, sesquiterpenoid and triterpenoid biosynthesis and isoquinoline alkaloid biosynthesis (Figure 7). Given that autumn leaf yellowing is correlated to ADB crown damage, SNPs within these functional groups may be a component of ash tolerance to *H. fraxineus*.

**Figure 8:**
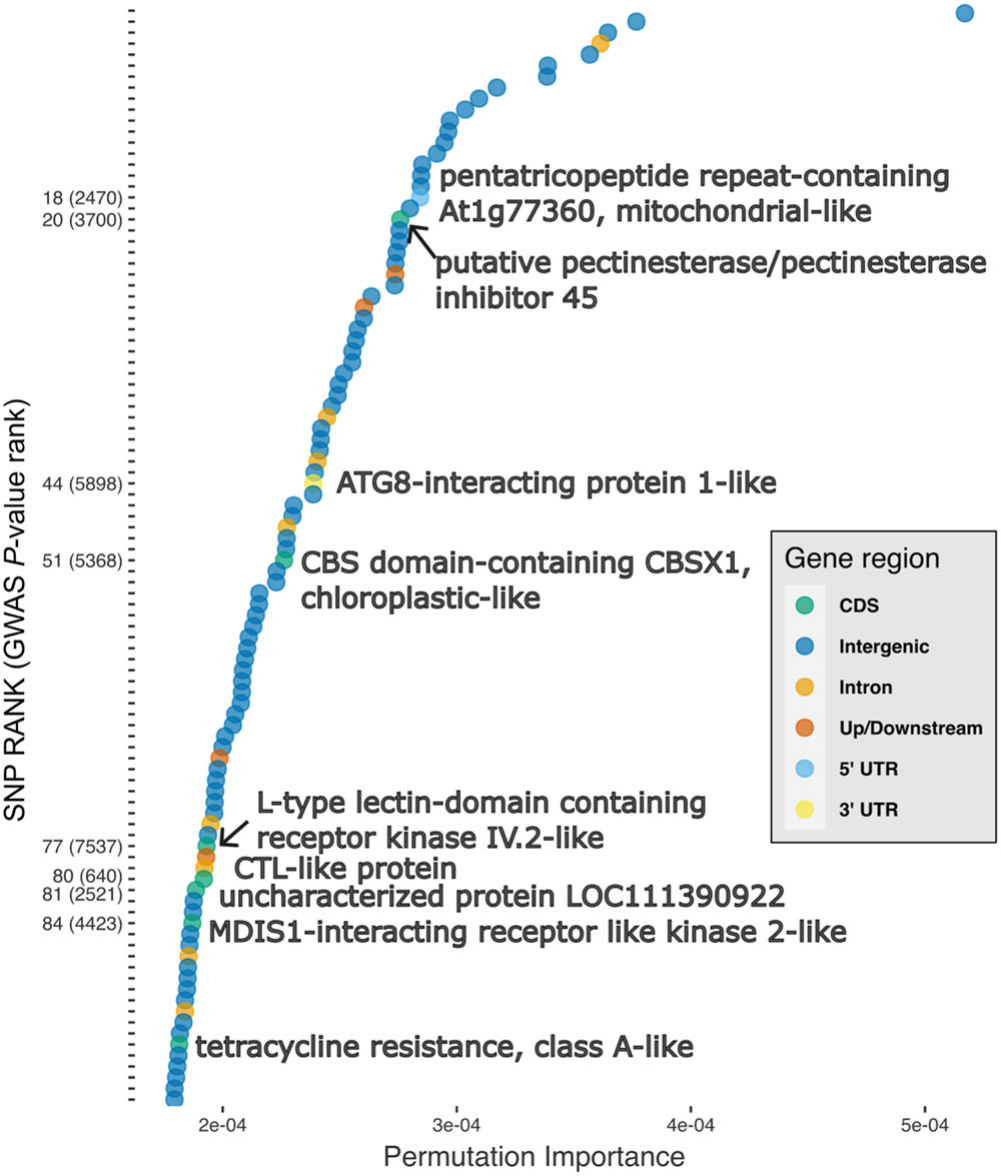
Top 100 SNPs linked to autumn leaf yellowing in *Fraxinus excelsior* ranked according to their permutation importance (PI) from a panel of 3740 SNPs. SNPs are marked according to their position within a genic region, SNPs within 500 bp of genic regions are marked ‘Up/Downstream’, SNPs resulting in a coding domain substitution (CDS) are shown in green and the value given in the *y* axis is their PI rank with GWAS rank in parentheses. SNPs which cause missense mutations have functionally annotated coding domains. Permutation importance score is shown in the *x* axis. [Color figure can be viewed at wileyonlinelibrary.com]

Within the top 100 SNPs with the highest PI, seven were within coding domains (where six produced non‐synonymous amino acid substitutions), two were in untranslated regions, four were up or downstream of a gene, eight were within introns and the remaining 79 were on intergenic regions (Figure 8). Of the seven SNPs on CDS, the first at SNP rank 20 was a putative pectinesterase with a pectin methylesterase inhibitor domain on the 5' region of the gene. Pectinesterase inhibitors have known anti‐virulence functions, as they mitigate the effect of pectin lyase effectors, which are a common component of the virulence arsenal in many plant pathogens (Dean et al. 2012; Liu et al. 2018). Two genes with non‐synonymous SNPs were located within gene regions for fungal elicitor recognition and are homologous to components of the PTI system (Jones and Dangl 2006). As previously described, PTI is an evolutionary conserved pathway which recognises molecular patterns and upon recognition activates defence components such as the oxidative burst. Specifically these are annotated as an *L*‐type lectin‐domain containing receptor kinase IV.2‐like, ranked at SNP 77 (Wang et al. 2014, 2018) and an MDIS1‐interacting receptor like kinase 2‐like ranked at SNP 84 (Coleman et al. 2021). The penultimate plant defence related SNP in a coding domain was ranked at 81 and has no characterised protein homologues, and the final SNP, ranked at 95 encodes a synonymous nucleotide on a coding domain (tetracycline resistance, class A‐like). There were two SNPs causing non‐synonymous amino acid substitutions on gene coding domains, which have functional homologues related to phenology regulation. The first of these at SNP rank 54 is within both a 3'UTR and a coding domain, which was functionally annotated as a homologue of CBSX protein which regulates flowering and leaf maturation through the thioredoxin system. The second amino acid substitution is within a choline transporter like‐1 (CTL‐1) which is associated to vesicular transport of the auxin transporter PIN1. Auxin is a plant hormone, which regulates many plant development functions including leaf abscission and yellowing (Jin et al. 2015).

## 4 Discussion

Here we found that associated SNPs could predict ash dieback crown damage with an adjusted R^2^ up to 63% based on 9155 unlinked SNPs. This does not suggest that the set of SNPs can predict the exact crown damage of a single tree from its genotype with such precision, because the phenotype of a given tree is dependent not only on its genotype but also on environmental variation. Results from common garden studies suggest moderate to high genetic narrow‐sense heritability (*h*_*ns*_^*2*^ = 0.37–0.72) (Pliura et al. 2011; Kjær et al. 2012; Lobo et al. 2014; Muñoz et al. 2016; Seidel et al. 2024) and broad sense heritability (*H*_*bs*_^2^ = 0.10–0.57) (McKinney et al. 2011; Stener 2013; Enderle et al. 2015; Seidel et al. 2024). The set of SNPs can only capture the genomic component of variation, which given this level of heritability will account for approximately 50% of the observed variation among trees, even in managed homogenous field trials. The phenotypes used in this study represent averages among replicates of the same genotype (or among siblings) grown in a common garden trial, thereby partly controlling for environmental effects, and it is encouraging that the associated SNPs could predict a high fraction of variation in crown damage scores.

Our findings suggest that ash dieback susceptibility is based on many genes with small effects, which is in line with GWAS results from Stocks et al. (2019) and Meger et al. (2024). However, there were no overlapping genes containing predictive or associated SNPs between this study and those of Stocks et al. (2019) and Meger et al. (2024). This may be due to geographic differences or factors such as the age of study trees and different phenotyping methods, as outlined in the introduction. We found no overlap between genes associated with crown damage level and autumn leaf yellowing – two quantitative genetically correlated traits. Although, this is potentially confounded by the impact of ash dieback on progression towards senescence; ash dieback causes defoliation, and therefore, the recording of leaf senescence (leaf loss in particular) is impacted by ADB (McKinney et al. 2012; Kirisits and Freinschlag 2012).

Our findings are important in relation to the likely dynamic of future host‐pathogen interaction, as tolerance controlled by a single gene or a few genes is more likely to be defeated compared to tolerance based on many genes (Ennos 2015). Despite the relatively low number of SNPs accounting for a large proportion of susceptibility in comparison to other polygenic traits identified in GWAS studies, it would take many evolutionary steps for the pathogen to overcome multiple tolerance loci, each with a small effect on the phenotype. Surpassing the tolerance offered by a single SNP would not make a large quantitative difference to the overall tree phenotype. Combined with recent results which present evidence that the biology of the pathogen does not favour selection of high virulence (Kosawang et al. 2020), and evidence of superior reproductive success of less susceptible individuals (Semizer‐Cuming et al. 2019, 2021), this gives some hope for the future of common ash. High mortality among the current generation is an acute concern but higher fitness of tolerant individuals, combined with restoration efforts and breeding programs, will strongly support the continued existence of *F. excelsior* in European forests.

Our findings are based on genotypes from several European trials where the genotypes have been carefully classified as superior or inferior (in terms of susceptibility to ADB), based on long‐term monitoring. We used a common protocol in data collection for the present study; trees with different genetic backgrounds were evaluated at different ages and sizes, and in trials with different growth conditions. Phenotypic measures were quantified as deviation from site average as individual trees were assessed at different ages and under different growth conditions. We assume that the average level of genetic tolerance is similar at the sample sites, which we consider a reasonable assumption with a recent emerging disease infecting a naïve host. However, phenology traits may be subject to local variation which may partly be lost, but this was unavoidable. Our finding – that we can predict the relative tolerance level of genotypes grown at different sites across countries and regions – support the robustness of the genomic predictions. Therefore, the outcome of the genome‐wide SNP analysis can be considered a robust prediction of ash dieback susceptibility across pan Central‐North European *F. excelsior* populations. It presents an important step towards understanding the genetic control of ash tree susceptibility in populations exposed to *H. fraxineus*. The results are therefore relevant and representative of the ash population across most of the disease outbreak area.

### 4.1 Genomic Background of Correlation Between Autumn Leaf Yellowing and ADB Susceptibility

Our results provide new insight into the intriguing strong quantitative genetic correlation previously reported from common garden studies, where early leaf yellowing was linked with lower levels of susceptibility to ADB (McKinney et al. 2011; Stener 2013). By assessing phenology and ADB susceptibility simultaneously, we confirmed the genotypic correlation between autumn leaf yellowing and ADB susceptibility assessed as crown damage score (Table 1). Moreover, we compared SNPs associated with each of the two correlated traits and found that a relatively small number of SNPs can also predict the genetic control in autumn leaf yellowing (e.g., adjusted *R* ^2^ of 33% with the 100 top ranked SNPs). Functional analysis of these SNPs revealed their enrichment of plant defence related genes (Figure 8). Predictive SNPs did not overlap (i.e., the SNPs were not the same) in the 100 top ranked SNPs in the correlated traits of ADB crown damage and autumn leaf yellowing. However, analysis of the molecular functions of the SNP loci suggested possible pleiotropic effects as for example defence components were associated with autumn leaf yellowing (Table 2).

### 4.2 Functional Analysis of Genes in Correlated Traits

We searched for SNP variants associated to correlated traits that offer indications on how trees avoid severe disease, that is, SNPs within functional regions which code for phenology and defence related genes. Within the autumn leaf yellowing trait, SNP variants code for amino acid substitutions in the regulation of flowering and leaf abscission (CBSX‐1), and a choline transporter like‐1 (CTL‐1) protein. These genes have homologues in plants which alter the timing of seasonal growth cycles (Wang et al. 2017; Murai et al. 2021). It is possible that the substitutions in these amino acids reflect observed variation in early change in autumn leaf colour as first reported by McKinney et al. (2011). Genetic correlation between phenotypic traits can either be a result of essentially the same genes controlling the traits (pleiotropy) or as a result of linkage disequilibrium (Lynch and Walsh 1998); in the latter case it must be explained by the evolutionary history or a bias created during selection of the tested genotypes.

Correlations between ADB crown damage and autumn leaf yellowing, together with highly predictive SNPs support the asynchronous growth theory, that is, trees avoid severe disease by early leaf abscission (McKinney et al. 2011; Harper et al. 2016). We did, however, not find a significant genetic correlation between ADB crown damage and autumn leaf loss, but as leaf loss is also a symptom of infection by *H. fraxineus*, leaf yellowing may be a more robust measure of autumn senescence, including shedding of leaves. The high level of genotypic correlation was based on genotypes selected before they were challenged by the novel pathogen (McKinney et al. 2011), and therefore selection bias is not likely to be an important explanation. Early shedding of leaves may create some level of disease escape, but our results suggest that tree defences are the major tolerance factor (Figures 3, 4, 5, 6 and Table 2). The data indicated that the two traits were controlled by different genes but still presented high genetic correlation. This suggests that genetic control of susceptibility is due to host defences and a possible coincidental relationship to autumn leaf yellowing. However, if the variation in susceptibility among individuals was caused only by autumn leaf yellowing, then the top ranked SNPs would be the same in ADB crown damage and autumn leaf yellowing. The view that active defence against the spread of the pathogen in host tissue is involved is also supported by the results from inoculations with *H. fraxineus* directly onto branches, where genotypes with low susceptibility to natural infections also showed limited bark necrosis development; furthermore development of necrotic bark lesions occurred much faster in genotypes with high susceptibility (McKinney et al. 2012), suggesting that an active defence against the spread of the pathogen is involved. As there is no co‐evolutionary history between *H. fraxineus* and common ash, it is likely to be a broad‐spectrum of defence mechanisms which limits the impact of *H. fraxineus* on tolerant ash genotypes.

### 4.3 SNPs in PRR Related Genes Are Linked to ADB Tolerance

Our results further show that standing genetic variation (i.e., the available allelic variation within a population at a given timepoint (Barrett and Schluter 2008) within tree defences is linked with variation in disease severity across the European ash population (Figure 4). This raises the possibility that individual variation in tolerance to ADB is mediated by host recognition of molecular patterns within *H. fraxineus*. Pattern recognition receptors (PRRs) are components of the plant defence system, and intriguingly, four of the six PRR related genes identified from top ranked SNPs are linked to autumn leaf yellowing. These PRR related genes are known to stimulate plant defence components (Boutrot and Zipfel 2017). As leaf yellowing is correlated with ADB crown damage, it may be evidence of gene pleiotropy (Table 2). As previously suggested, an active defence is likely to be a key component of tolerance to ADB, and recognition of pathogen components of the hemibiotroph *H. fraxineus* by *F. excelsior* is a possible mechanism of tolerance to ADB (Lobo et al. 2015; Nielsen et al. 2017; Mansfield et al. 2018). Here, we provide putative genomic evidence of ADB tolerance among individuals through pattern triggered immunity mediated recognition of an external threat and a response to that threat through defence activation.

## 5 Conclusions

This study highlights the genetic architecture and possible mechanisms involved in the genetic control over ADB crown damage and phenology traits. The implications of these findings are: (1) ADB tolerance is polygenic, but a large proportion of the variance can be predicted by a relatively small number of SNPs (Figure 4); (2) At the gene level, there is evidence of disease tolerance via plant defence recognition of *H. fraxineus*; (3) Phenological avoidance of severe disease is a component of tolerance, especially as it is correlated with ADB crown damage. However, a previous study showed that direct inoculation with *H. fraxineus* and the resultant defence response is responsible for individual variation to the pathogen (Lobo et al. 2015).

Our findings highlight that ADB susceptibility is determined by combinations of polygenic traits. When assessing crown damage levels, we see the combined effect of different mechanisms, that is, disease tolerance (host trees limiting the growth of the pathogen) and disease escape (autumn leaf yellowing, as a proxy for senescence) but possibly also other mechanisms. A tree that is not able to limit the growth of the pathogen (i.e., potentially showing low tolerance) might still be able to escape the disease due to early senescence (as indicated by autumn leaf yellowing) leading to premature leaf shed or perhaps pathogen growth is inhibited in yellow leaves, and hence might show limited crown damage. This emphasises the difficulty in identifying relevant genes in GWAS using crown damage levels or tree health as phenotypic traits. In the future, a better understanding of the molecular defence mechanisms in ash trees in response to the pathogen are needed. One way to approach this might be to target more specific traits, e.g. necrosis length after controlled inoculations might provide a better estimate of disease tolerance which is not confounded with disease escape. However, in the end the complex interplay of different mechanisms determines the survival of ash trees.

## Supporting information

Supplementary information.

Supplementary information.

Supplementary information.

Supplementary information.

Supplementary information.

Supplementary information.

Supplementary information.

## Data Availability

The data that support the findings of this study are openly available in NCBI BioProject at https://www.ncbi.nlm.nih.gov/bioproject?term=PRJNA1055085&cmd=DetailsSearch&log$=activity, reference number PRJNA1055085.
