# Supplementary material for: Multiple, Single Trait GWAS and Supervised Machine Learning Reveal the Genetic Architecture of Fraxinus excelsior Tolerance to Ash Dieback in Europe
Source: Plant Cell Environ. 2025 Jan 17;48(5):3793–809. doi: 10.1111/pce.15361 (PMC11963480; doi:10.1111/pce.15361)
Supplement: Supplementary file 1 — Supplementary information. [file PCE-48-3793-s007.pdf]

Phenology scores of ash

0

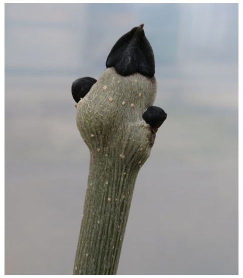

Vinterstadium

Winter stage
Dormant buds

1

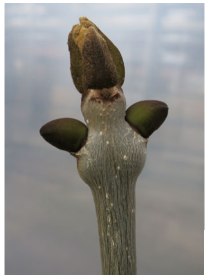

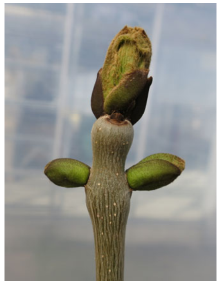

Svulmende knopper, knopskæl omlutter blade helt

Swollen bud, Bud scales enclose leaves entirely

2

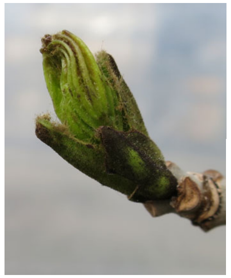

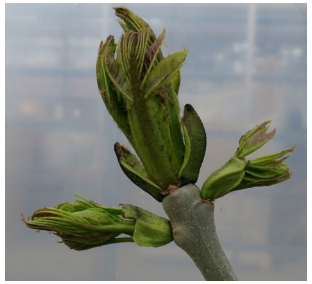

Knopper bryder, blade (akkurat) synlige

Buds are bursting, leaves (just) visible

3

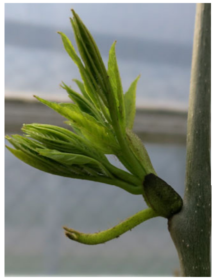

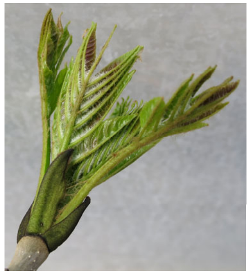

Meget små blade, netop undsluppet knoppen

Leaves very small,
Just escaped the bud

4

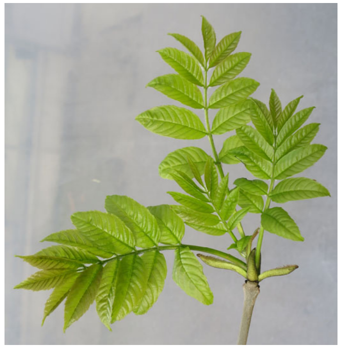

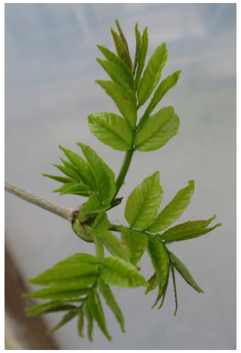

Små udfoldede blade, i begyndelsen af strækningsfasen

Leaves open and glossy, shoots starting to stretch

5

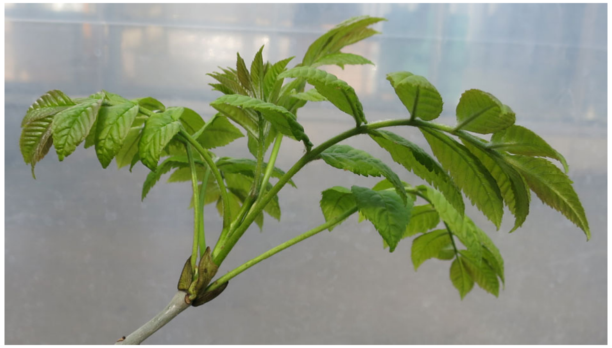

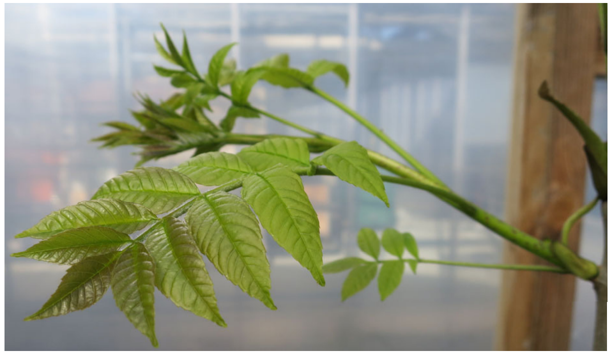

Blade i fremskreden strækningsfase. Stadig skinnende

Leaves stretched markedly, still glossy

6

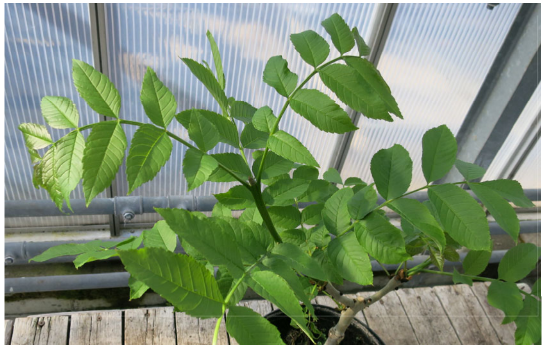

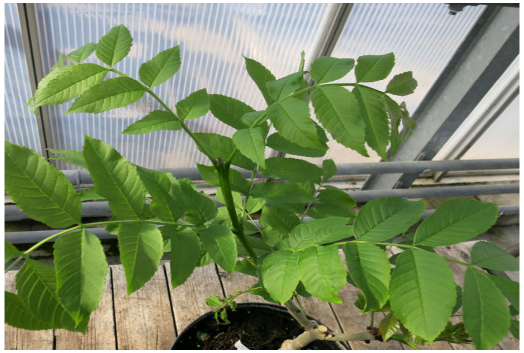

Størrelsesmæssigt færdigtudviklede blade, stadigt vårfriske, begynder at hærde.

Leaves full size, still "spring fresh", not completely hardened

7

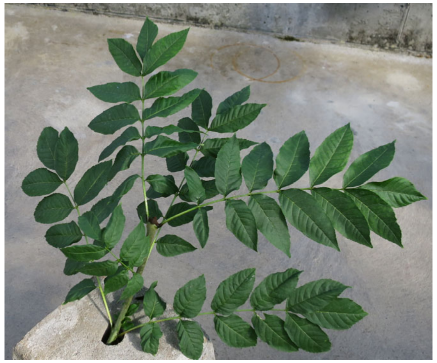

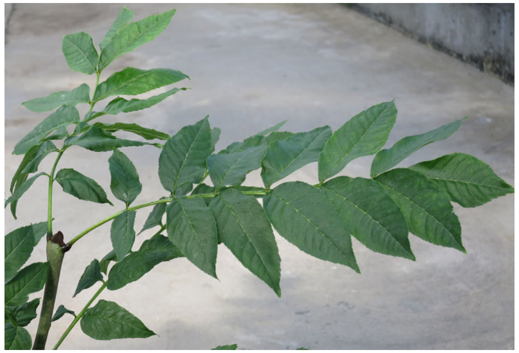

Færdigtudviklede helt hærdede blade.

Mature leaves, all leaves are dim and hardened
