# Supplementary material for: Multiple, Single Trait GWAS and Supervised Machine Learning Reveal the Genetic Architecture of Fraxinus excelsior Tolerance to Ash Dieback in Europe
Source: Plant Cell Environ. 2025 Jan 17;48(5):3793–809. doi: 10.1111/pce.15361 (PMC11963480; doi:10.1111/pce.15361)
Supplement: Supplementary file 2 — Supplementary information. [file PCE-48-3793-s001.pdf]

Senescence scoring 13/9-2019

Fraxinus mandshurica, F. angustifolia and F. excelsior

Lene Rostgaard Nielsen

Examples of senescence scoring of Fraxinus mandshurica, F. angustifolia and F. excelsior from the arboretum in Hørsholm, Denmark. Scored by Lars Nørgaard Hansen, Kristina Thomas and Lene R. Nielsen on September 13 2019.

The following descriptions and photos are to illustrate the three different scores that were used in the project "AshAdapt" to evaluate the senescence stage of trees at a certain time point. Here, the trees (different species of ash) were examined in the arboretum, Hørsholm, Denmark, on September 13 2019. Assessors were Lars Nørgaard Hansen, Kristina Thomas (visiting from Germany) and me. Shading of trees in the arboretum varied making it difficult to compare the photos.

1. **<u>Autumn leaf yellowing</u> evaluates the autumn colouring of the leaves**. The score reflects the average performance of the tree.

Score	
0	Dark green
1	Dark green with yellowing leaf nerves
2	Green with yellow spots on leaflets
3	Yellowing leaflets
4	Completely yellow leaves

0	1	2	3	4

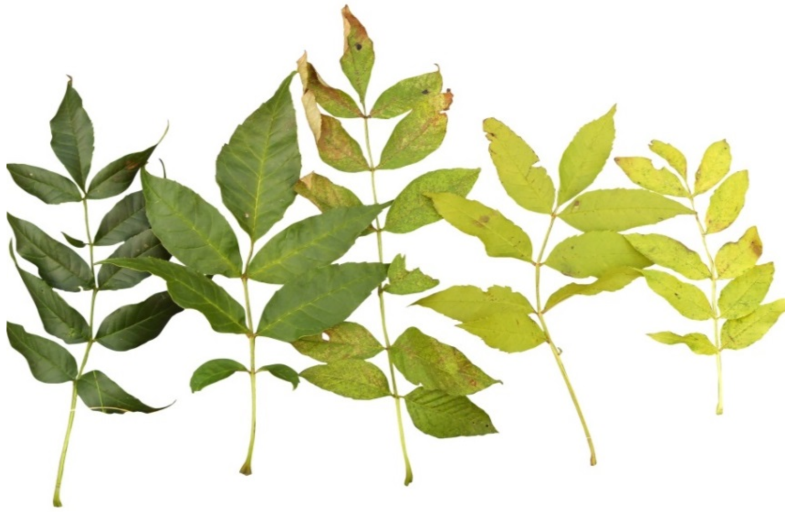

2. **<u>Autumn leaf loss</u> is the percentage of the foliage (in classes) that has been shed presumably due to senescence.** We try to exclude leaf loss that is clearly an effect of ash dieback (branches that look dead due to disease). The whole living crown (primary crown and epicormic shoots) are taken into account.

0	No leaf shed detected	
1	0-10 %	<10 %
2	11-20 %	<20 %
3	21-30%	<30%
4	31-40%	<40%
5	41-50%	<50%
6	51-60%	<60%
7	61-70%	<70%
8	71-80%	<80%
9	81-90%	<90%
10	91-99 %	<99 %
(11	No leaves left)	

3. **<u>Autumn status</u> reflects the overall autumn senescence stage of the tree based on the remaining leaves in the crown**. Symptoms of senescence of foliage are: summer green colour becomes lighter, yellowing, brownish, withering (typically from the rim towards the middle) and crusted, dried out, or hanging leaves. Thus, the score is gradual and shows average tree senescence level (full dormancy = 100%). NB! Average performance meaning that a tree showing prominent symptoms in a small part of the crown may score the same as a tree showing milder symptoms but in a larger part of the crown.

0	All remaining leaves still summer green
1	Remaining crown shows 1-10% dormancy
2	Remaining crown shows 11-20% dormancy
3	Remaining crown shows 21-30% dormancy
4	Remaining crown shows 31-40% dormancy
5	Remaining crown shows 41-50% dormancy
6	Remaining crown shows 51-60% dormancy
7	Remaining crown shows 61-70% dormancy
8	Remaining crown shows 71-80% dormancy
9	Remaining crown shows 81-90% dormancy
10	Remaining crown shows 91-99% dormancy (highly pronounced senescence symptoms - almost dormant)
(11	Complete senescence -All remaining leaves dead - 100% dormancy)

Examples illustrated with photos

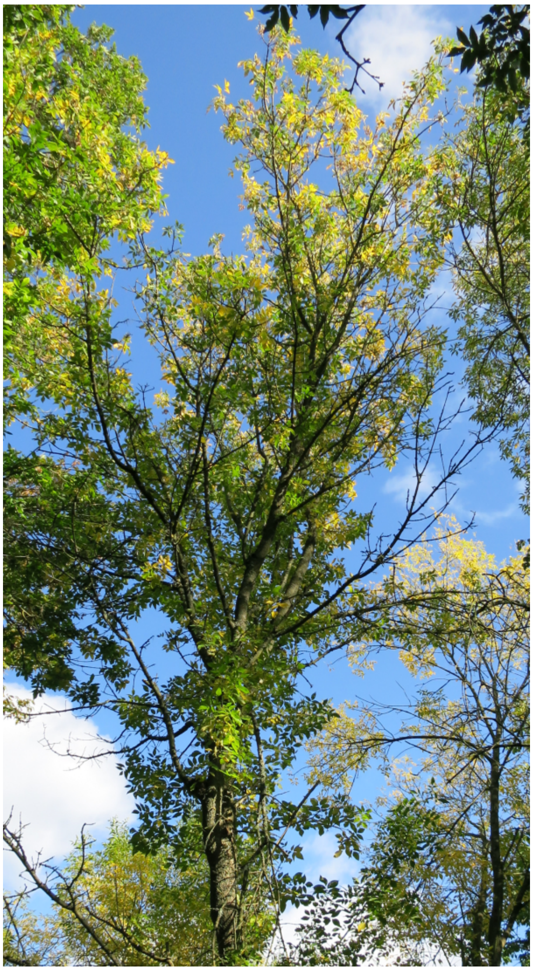

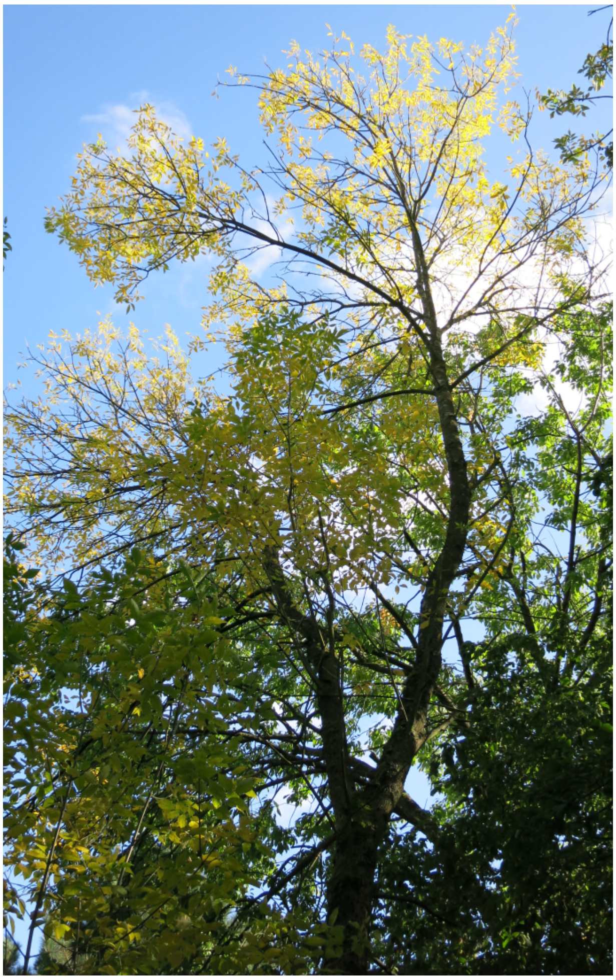

Autumn leaf yellowing: 3
Autumn leaf loss: 2
Autumn status: 6

Autumn leaf yellowing: 4
Autumn leaf loss: 5
Autumn status: 9

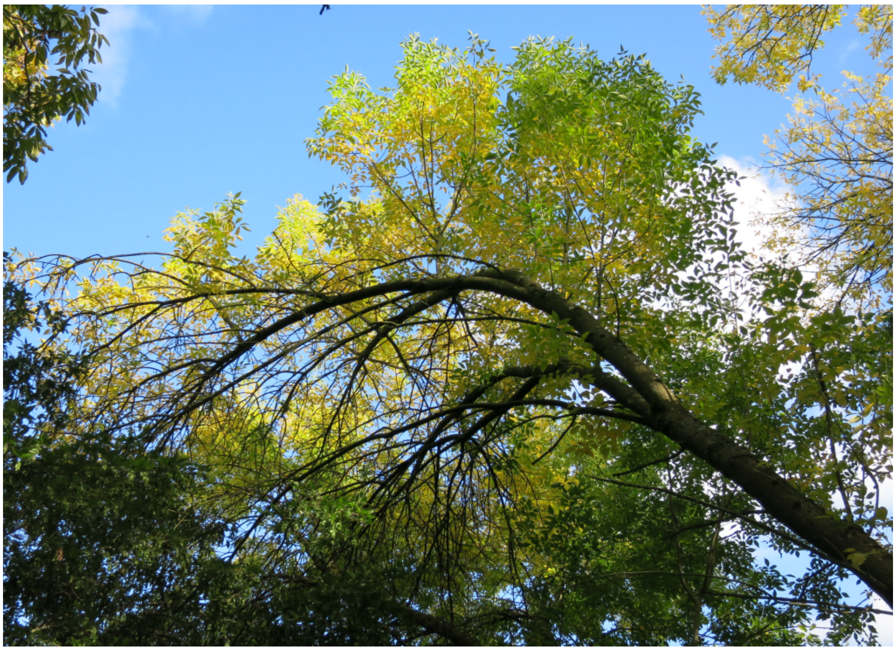

Autumn leaf yellowing: 3
Autumn leaf loss: 1
Autumn status: 7

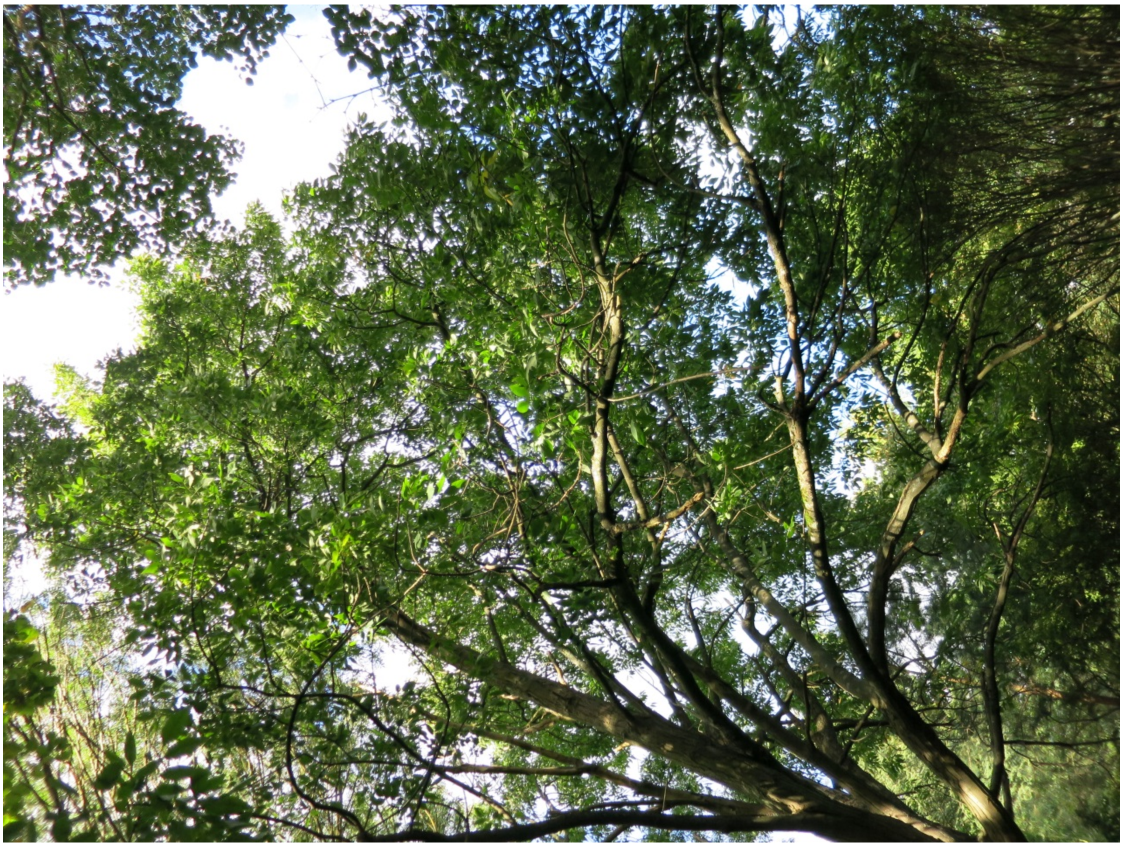

Autumn leaf yellowing: 0
Autumn leaf loss: 0
Autumn status: 1

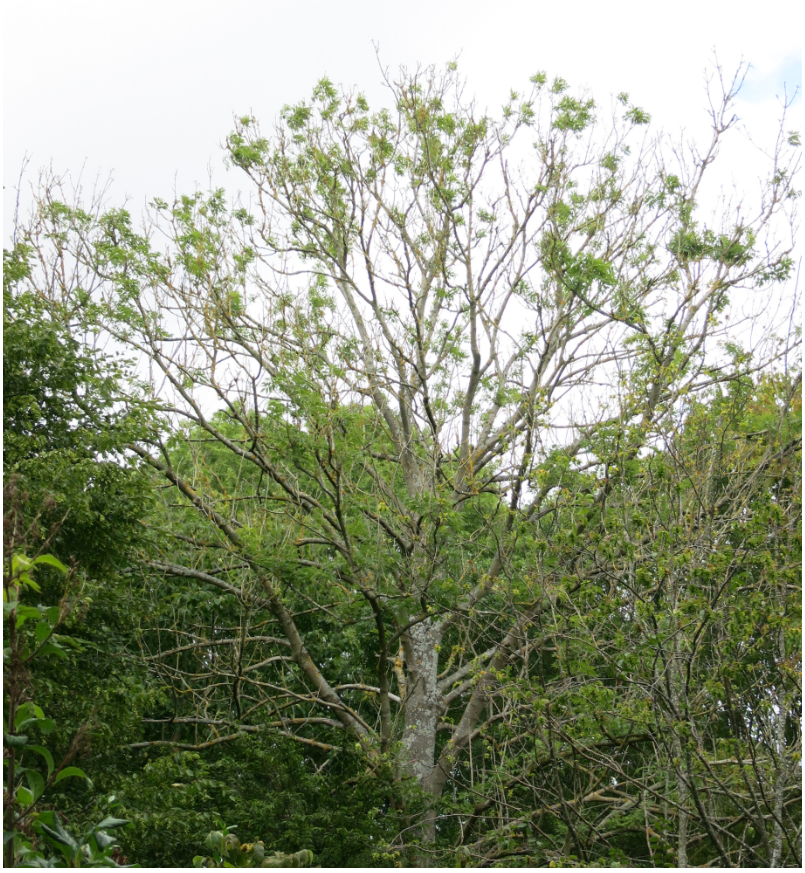

Autumn leaf yellow: 0
Autumn leaf loss: 0
Autumn status: 0
NB Sick tree!

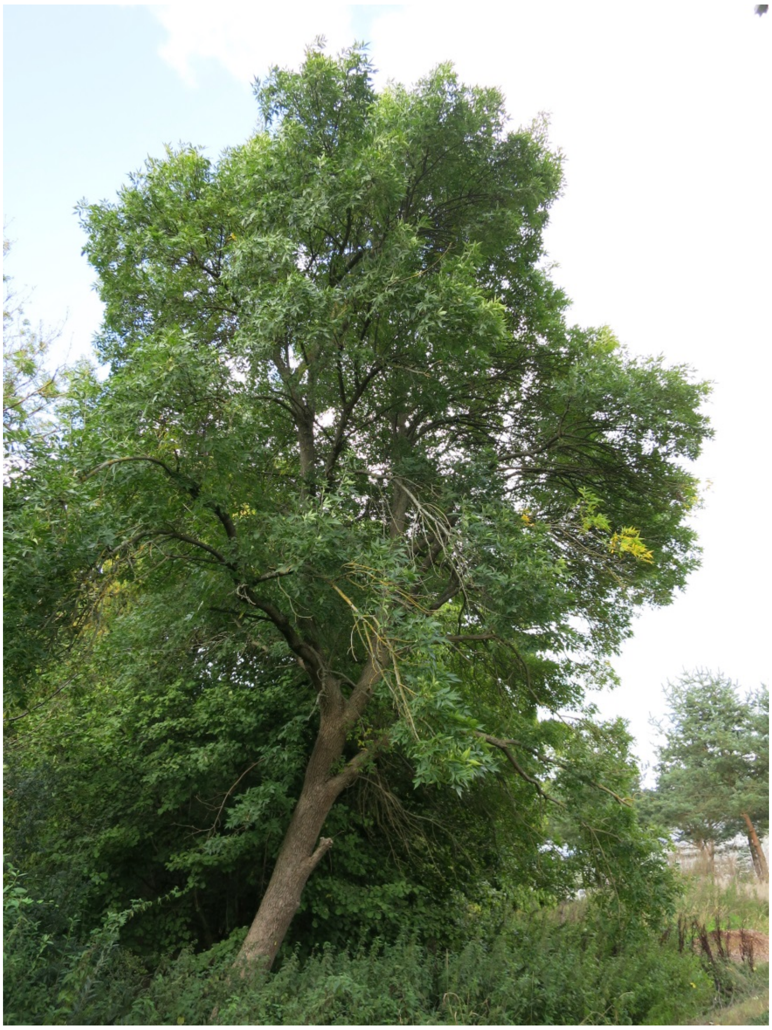

Autumn leaf yellowing: 0
Autumn leaf loss: 1
Autumn status: 1

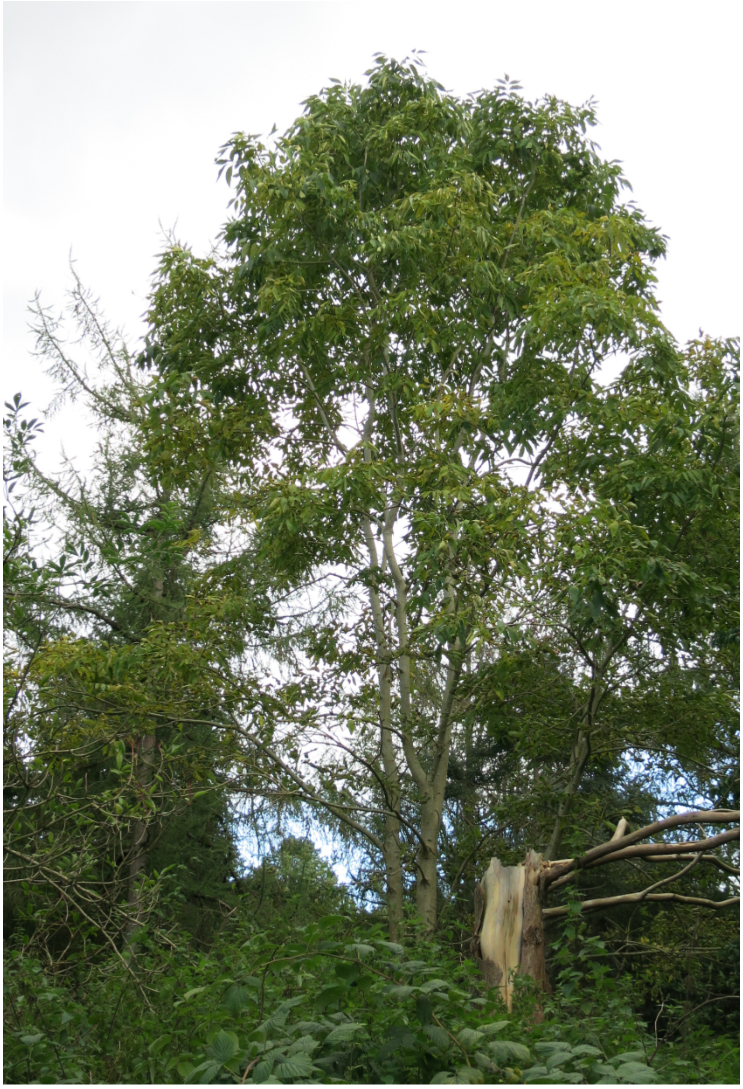

Autumn leaf yellowing: 2
Autumn leaf loss: 2
Autumn status: 4

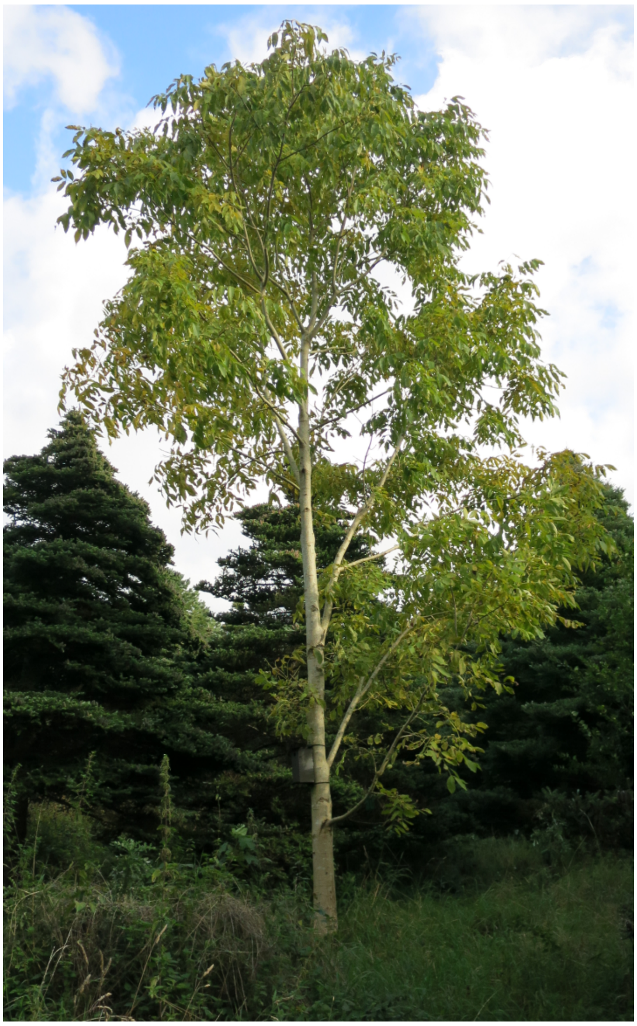

Autumn leaf yellowing: 2
Autumn leaf loss: 1
Autumn status: 6

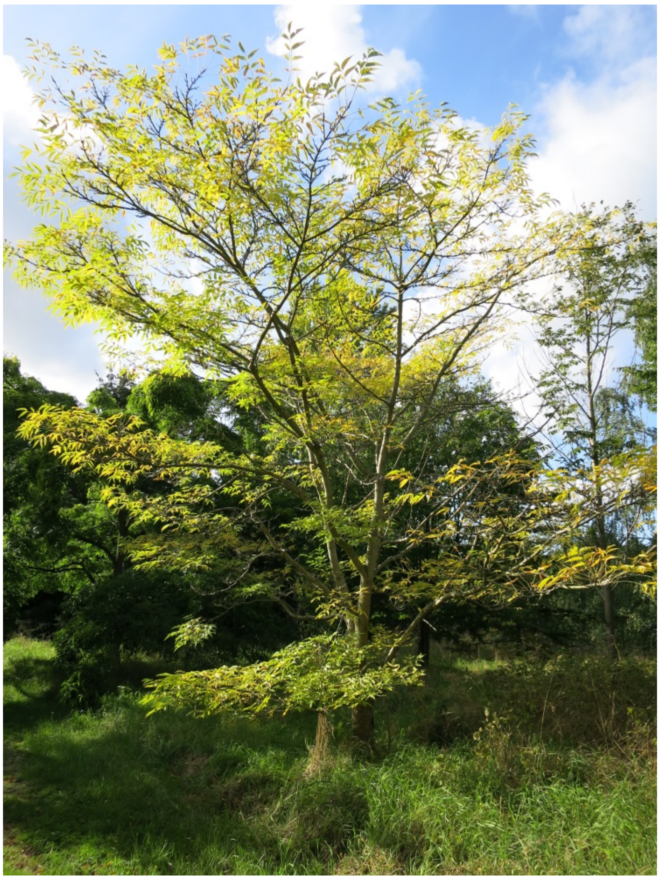

Autumn leaf yellowing: 3
Autumn leaf loss: 4
Autumn status: 8

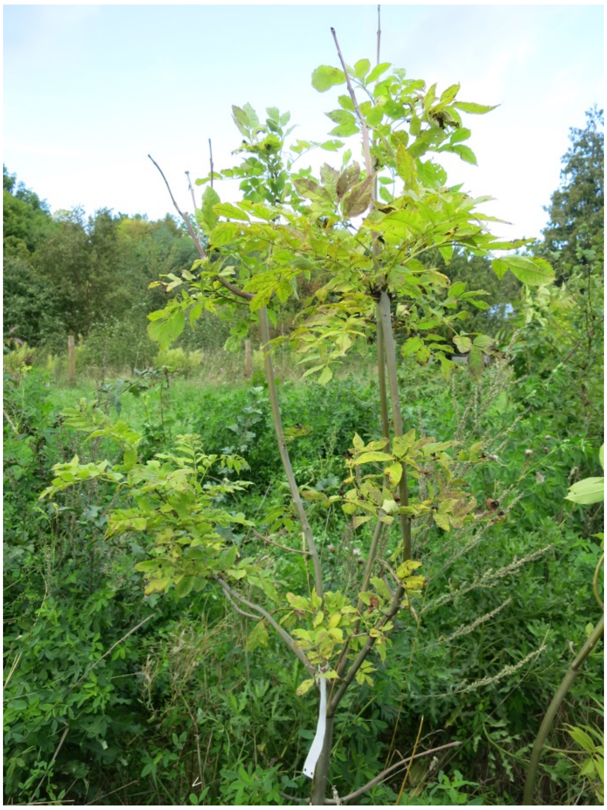

Autumn leaf yellowing: 2
Autumn leaf loss: 0
Autumn status: 7

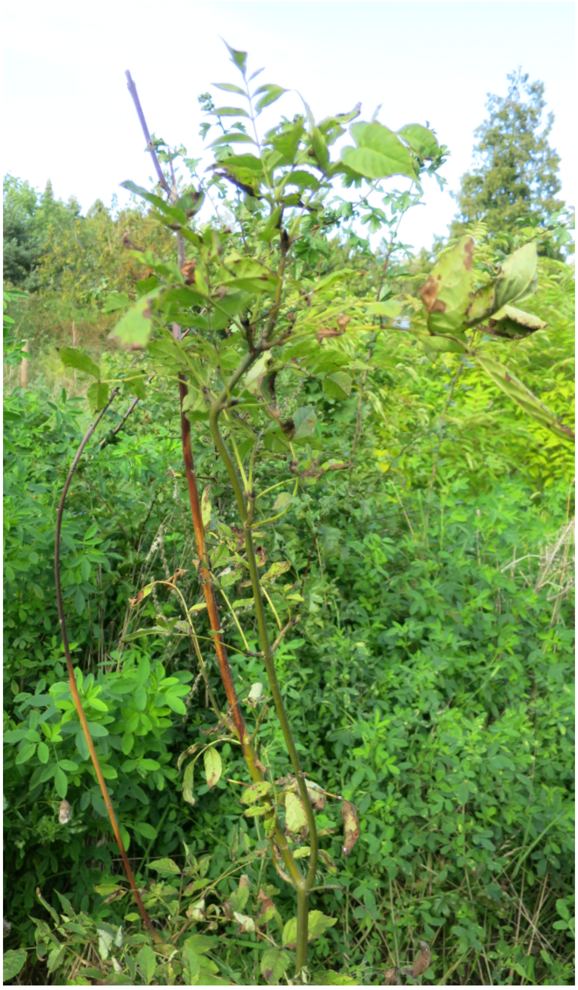

Autumn leaf yellowing: 2
Autumn leaf loss: 1
Autumn status: 6

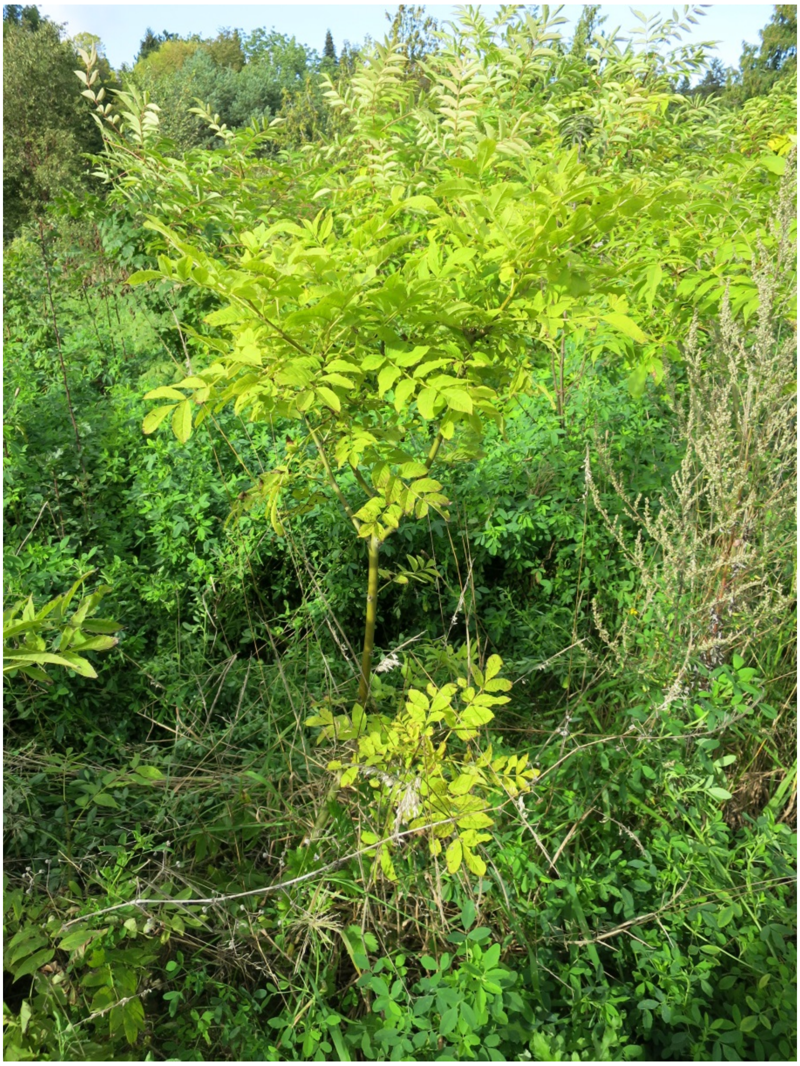

Autumn leaf yellowing: 3
Autumn leaf loss: 0
Autumn status: 7
